# Brian2CUDA: Flexible and Efficient Simulation of Spiking Neural Network Models on GPUs

**DOI:** 10.3389/fninf.2022.883700

**Published:** 2022-10-31

**Authors:** Denis Alevi, Marcel Stimberg, Henning Sprekeler, Klaus Obermayer, Moritz Augustin

**Affiliations:** ^1^Technische Universität Berlin, Chair of Modelling of Cognitive Processes, Berlin, Germany; ^2^Bernstein Center for Computational Neuroscience Berlin, Berlin, Germany; ^3^Sorbonne Université, INSERM, CNRS, Institut de la Vision, Paris, France; ^4^Technische Universität Berlin, Chair of Neural Information Processing, Berlin, Germany

**Keywords:** spiking neural networks, simulator, GPU, CUDA, Python, software, open-source, parallel algorithm

## Abstract

Graphics processing units (GPUs) are widely available and have been used with great success to accelerate scientific computing in the last decade. These advances, however, are often not available to researchers interested in simulating spiking neural networks, but lacking the technical knowledge to write the necessary low-level code. Writing low-level code is not necessary when using the popular Brian simulator, which provides a framework to generate efficient CPU code from high-level model definitions in Python. Here, we present Brian2CUDA, an open-source software that extends the Brian simulator with a GPU backend. Our implementation generates efficient code for the numerical integration of neuronal states and for the propagation of synaptic events on GPUs, making use of their massively parallel arithmetic capabilities. We benchmark the performance improvements of our software for several model types and find that it can accelerate simulations by up to three orders of magnitude compared to Brian's CPU backend. Currently, Brian2CUDA is the only package that supports Brian's full feature set on GPUs, including arbitrary neuron and synapse models, plasticity rules, and heterogeneous delays. When comparing its performance with Brian2GeNN, another GPU-based backend for the Brian simulator with fewer features, we find that Brian2CUDA gives comparable speedups, while being typically slower for small and faster for large networks. By combining the flexibility of the Brian simulator with the simulation speed of GPUs, Brian2CUDA enables researchers to efficiently simulate spiking neural networks with minimal effort and thereby makes the advancements of GPU computing available to a larger audience of neuroscientists.

## 1. Introduction

In computational neuroscience, there is high demand for computationally efficient simulations allowing for realtime applications or exhaustive parameter explorations. Efficient simulations require both optimized simulation software and powerful hardware. In practice, there is always a trade-off between the performance of the hardware and its price and accessibility. A promising technology with a very beneficial performance–cost trade-off are graphics processing units (GPUs) with their massively parallel arithmetic capabilities. While they were initially designed for computer graphics, they have since become commonly used for general-purpose computing, leading to their designation as general-purpose graphics processing units (GPGPUs). The most popular framework is the Compute Unified Device Architecture (CUDA; NVIDIA Corporation, 2007–2022) which allows users to write parallel code for GPUs in an extension of the C/C++ programming languages. To make efficient use of GPUs, simulation code has to perform computations in a highly parallel way. This parallelization is rather straightforward to implement for some aspects of neuronal models, e.g., the numerical integration of neuronal state variables over a simulation time step, but is non-trivial for other aspects, e.g., spike propagation with synaptic delays (cf. Brette and Goodman, 2012).

The earliest attempts at using the GPU (e.g., Bernhard and Keriven, 2006; Nageswaran et al., 2009) explored the general feasibility of accelerating simulations of spiking neural networks, and described many of the challenges that are still relevant today. To benefit from the capabilities of a GPU, a simulation needs to be parallelized efficiently, parallel memory access to shared memory has to be handled carefully, and synaptic connections have to be stored in sparse data structures to fit into the limited memory of GPUs (Nageswaran et al., 2009). The earliest implementations were typically technology demonstrations, but not released as software packages to be used by other researchers. This changed in the following years, as a number of general-purpose simulators such as NEMO (Fidjeland et al., 2009; Fidjeland and Shanahan, 2010), CNS (Mutch et al., 2010), CARLsim (Richert et al., 2011; Chou et al., 2018), and NCS6 (Hoang et al., 2013) were released. While these simulators could be adapted to a researcher's needs, they typically only supported specific neuron models or network structures: The NEMO, CARLsim, and NCS6 simulators were built to simulate networks of leaky integrate-and-fire or quadratic integrate-and-fire model (Izhikevich, 2003) neurons, and the CNS simulator was built to simulate networks structured in cortical layers. Extending these simulators to other models requires a researcher to write CUDA code and is therefore not accessible to many researchers without the necessary technical background.

Most recent simulators (e.g., Abi Akar et al., 2019; Panagiotou et al., 2021; Ben-Shalom et al., 2022) do not come with predefined neuron models, but instead translate neuron model definitions created for the NEURON simulator (Carnevale and Hines, 2006) or model definitions exported to NeuroML (Cannon et al., 2014) by a compatible simulator. An advantage of this approach is that it makes it possible to immediately reuse a large number of existing neuron models. On the other hand, this workflow is not ideal for researchers that want to adapt and change existing models, or introduce completely new ones. For these use cases, the fact that the model description and its simulation require more than one software package can be a major hurdle.

A number of simulators addressed this issue by using code generation (Goodman, 2010; Blundell et al., 2018). In such a framework, the model description in a convenient high-level or domain-specific language is an integral part of the simulator itself. When starting a simulation, these model descriptions are translated into efficient low-level code, compiled, and executed. Two simulators that have used this approach to generate code for GPUs are ANNarchy (Vitay et al., 2015), which specializes in networks that mix rate and spike-based elements, and GeNN (Yavuz et al., 2016), where model descriptions have to be specified in a variant of C++ (but note that the main simulation code can be written as a Python script *via* the PyGeNN interface; Knight et al., 2021b).

The Brian^1^ simulator (Stimberg et al., 2019a) is a widely used neural simulator that provides a user-friendly system for model descriptions based on mathematical equations, as well as an extensible code generation framework. So far, this framework was only capable of generating C++ code for multithreaded execution on central processing units (CPUs). Recently, Brian's framework has been extended to generate code for the GeNN simulator (Brian2GeNN; Stimberg et al., 2020b), making it finally possible to run Brian simulations on the GPU. However, this approach limits simulations to the common feature set provided by Brian, GeNN, and the Brian2GeNN interface: some of Brian's features (e.g., multicompartmental models) are not supported by GeNN at all, and the support for other features (e.g., heterogeneous synaptic delays) was added after the creation of the Brian2GeNN interface, and they are therefore also unsupported at this time.

Here, we present a new approach to GPU code generation with the Brian simulator. This interface, named Brian2CUDA, directly generates CUDA code for the GPU, and supports the full set of features that the Brian simulator offers. It can therefore be used as a drop-in replacement in all situations where multithreaded CPU code generation was used previously, including simulations of detailed network models of neurons, synapses, and glia cells (Stimberg et al., 2019b), or when optimizing neuronal models with the brian2modelfitting toolbox (Teska et al., 2020).

We describe how our approach exploits non-trivially parallelizable simulation parts, in particular the data structures and algorithms for the propagation of neuronal spikes through a network taking into account – potentially hetereogeneous – synaptic delays. For several relevant generic model classes, we compare the performance of Brian2CUDA with Brian's built-in multithreaded execution on CPUs and – where possible – with the Brian2GeNN interface. The results show that Brian2CUDA strongly outperforms the multithreaded execution on CPUs, sometimes by orders of magnitudes. Its performance is comparable to the performance of Brian2GeNN. For large networks, Brian2CUDA is faster, while for smaller networks slightly slower.

Our code is available as open source software under a free license at GitHub: https://github.com/brian-team/brian2cuda.

## 2. Method

Brian2CUDA implements a new Brian backend, which runs spiking neural network simulations on NVIDIA graphics processing units (GPUs). It makes use of Brian's code generation system to generate C++/CUDA code based on a user's model definition in Python.

In the following, we provide in Section 2.1 background on the Brian simulator and describe how our proposed CUDA backend can be used. In Section 2.2, we outline GPU programming essentials. Section 2.3 contains the algorithms implemented in Brian2CUDA including neuronal state updates, spike propagation, and synaptic effect application. Section 2.4 summarizes the alternative CUDA-based simulator Brian2GeNN and in Section 2.5 we specify the benchmark models and experimental procedure.

### 2.1. Brian Simulation and Code Generation

**Figure 1:**
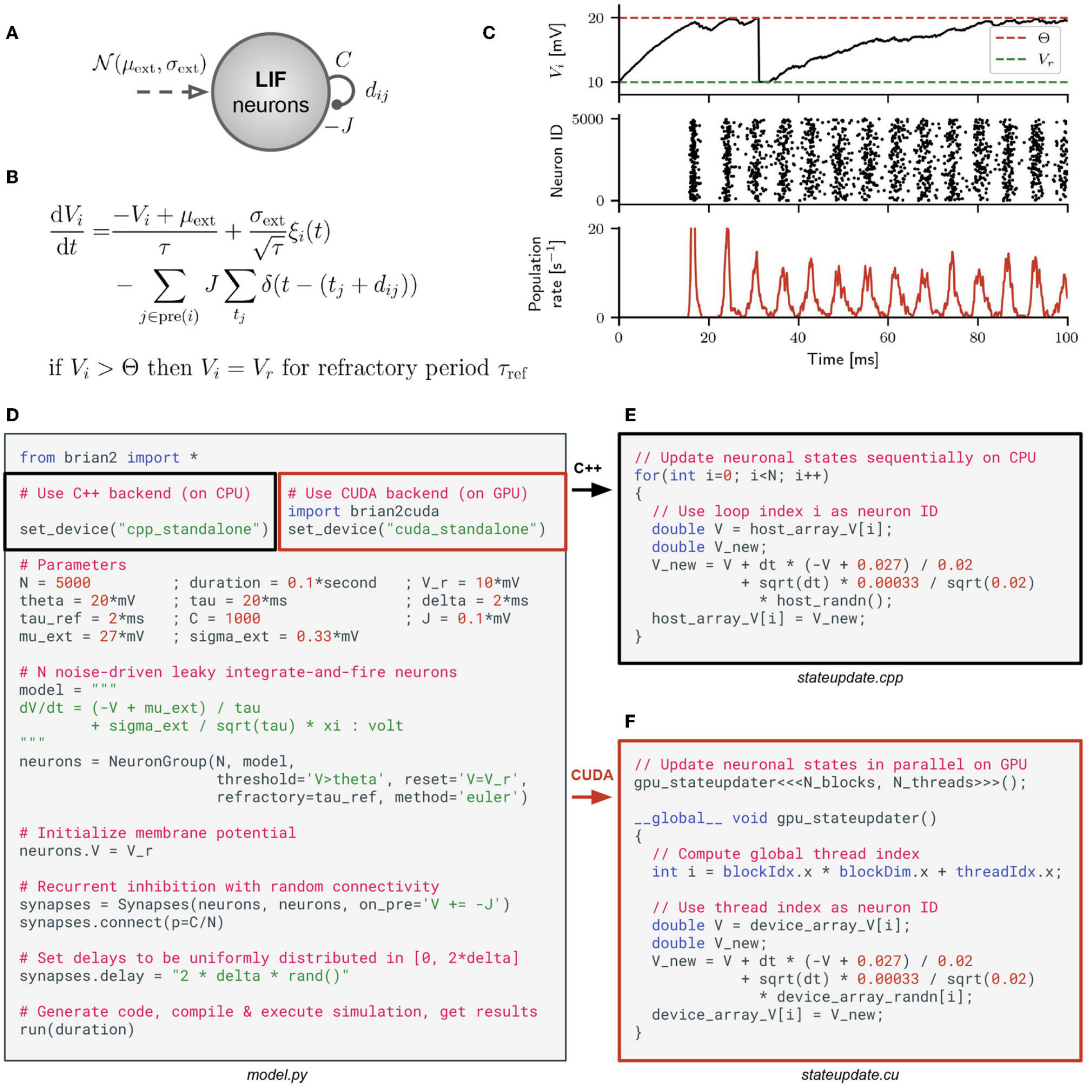
Brian model definition and C++/CUDA code generation. **(A)** Population of leaky integrate-and-fire (LIF) neurons with recurrent inhibitory coupling *J*, average number of random synapses per neuron *C* and synaptic transmission delays *d*_*ij*_ between neurons *j* and *i*. The neurons are driven by external Gaussian white noise with mean μ_ext_ and standard deviation σ_ext_ (model from Brunel and Hakim, 1999). **(B)** Corresponding stochastic differential equation defining the dynamics of the membrane potential *V*_*i*_ of a single LIF neuron *i* [with *i* = 1, …, *N*; membrane time constant τ; unit Gaussian white noise process ξ_*i*_(*t*) that is uncorrelated across neurons; *j* ∈ pre(*i*) runs over all neurons *j* that are presynaptic to neuron *i*; *t*_*j*_ are all spike times of neuron *j*; Dirac delta function δ(*x*)]. When the voltage *V*_*i*_ crosses threshold Θ, the neuron spikes and is set to the reset voltage *V*_r_ for a refractory period τ_ref_. **(C)** Network dynamics from simulating the model with *N* = 5000 leaky integrate-and-fire (LIF) neurons in Brian. Top panel: voltage trace for one exemplary neuron *i*. Middle panel: raster plot of the spike times for all neurons in the network. Bottom panel: instantaneous mean firing rate across all neurons. **(D)** A Python script implementing the model in Brian, either with its C++ backend (black blox) or with Brian2CUDA's CUDA backend (red box). In this example, the synaptic transmission delays are independently sampled from a uniform distribution $d_{ij}\sim U\left(0,4\right)\text{ms}$. **(E)** Simplified version of generated C++ code to update all neuronal states defined by the voltages *V*_*i*_ when using the C++ backend in Brian. **(F)** The same for the CUDA backend in Brian2CUDA. Here the CUDA kernel gpu_stateupdater is launched with *N*_blocks_ × *N*_threads_ parallel threads.

Brian is a simulator for spiking neural networks written in Python (Stimberg et al., 2014, 2019a). It is designed to be highly flexible and easy to use by using its own domain language to define models. This allows users to define arbitrary differential equations in Python strings. As an example, consider the model (Brunel and Hakim, 1999) depicted in Figure 1A. It consists of a population of *N* leaky integrate-and-fire (LIF) neurons with sparse random recurrent inhibitory connections, which are driven by Gaussian white noise. This model can be described by the differential equation depicted in Figure 1B. Since the inhibitory feedback is strong enough, the model exhibits fast global oscillations in the population firing rate while the single neuron firing rates remain small (see Figure 1C). A Python script that implements this model in Brian is shown in Figure 1D. By changing two lines of code, the simulation can be switched from Brian's C++ backend to our new Brian2CUDA backend.

Both backends generate simulation code in their target language (C++ or CUDA) which is then compiled and executed. The generated code implements the simulation loop, memory management, and all computations; it can be executed independently of Python. Figure 1E illustrates the Brian C++ backend, showing a simplified example of the generated C++ code for updating all neuronal states at a single time step of the simulation. In our example, one central processing unit (CPU) thread sequentially updates the membrane voltage *V*_*i*_ for each neuron *i*. To speed up simulations, the Brian C++ backend can be configured to use OpenMP to parallelize computations over multiple CPU threads (not shown here). Our Brian2CUDA backend extends the C++ backend to generate C++/CUDA code. The simulation loop and memory management are implemented in C++ and executed on a single CPU thread, while most computations are implemented in CUDA and are parallelized on the GPU. Figure 1F shows the same neuronal state updates as before, but now implemented in CUDA. The voltages of all neurons are updated in parallel by all available threads on a GPU.

### 2.2. GPU Programming With CUDA

To implement software that runs on NVIDIA GPUs, the Compute Unified Device Architecture (CUDA) programming model is used. CUDA works with multiple programming languages, and here we use the CUDA API implemented in C++.

#### 2.2.1. CUDA Programming Logic

##### 2.2.1.1. Thread Hierarchy

**Figure 2:**
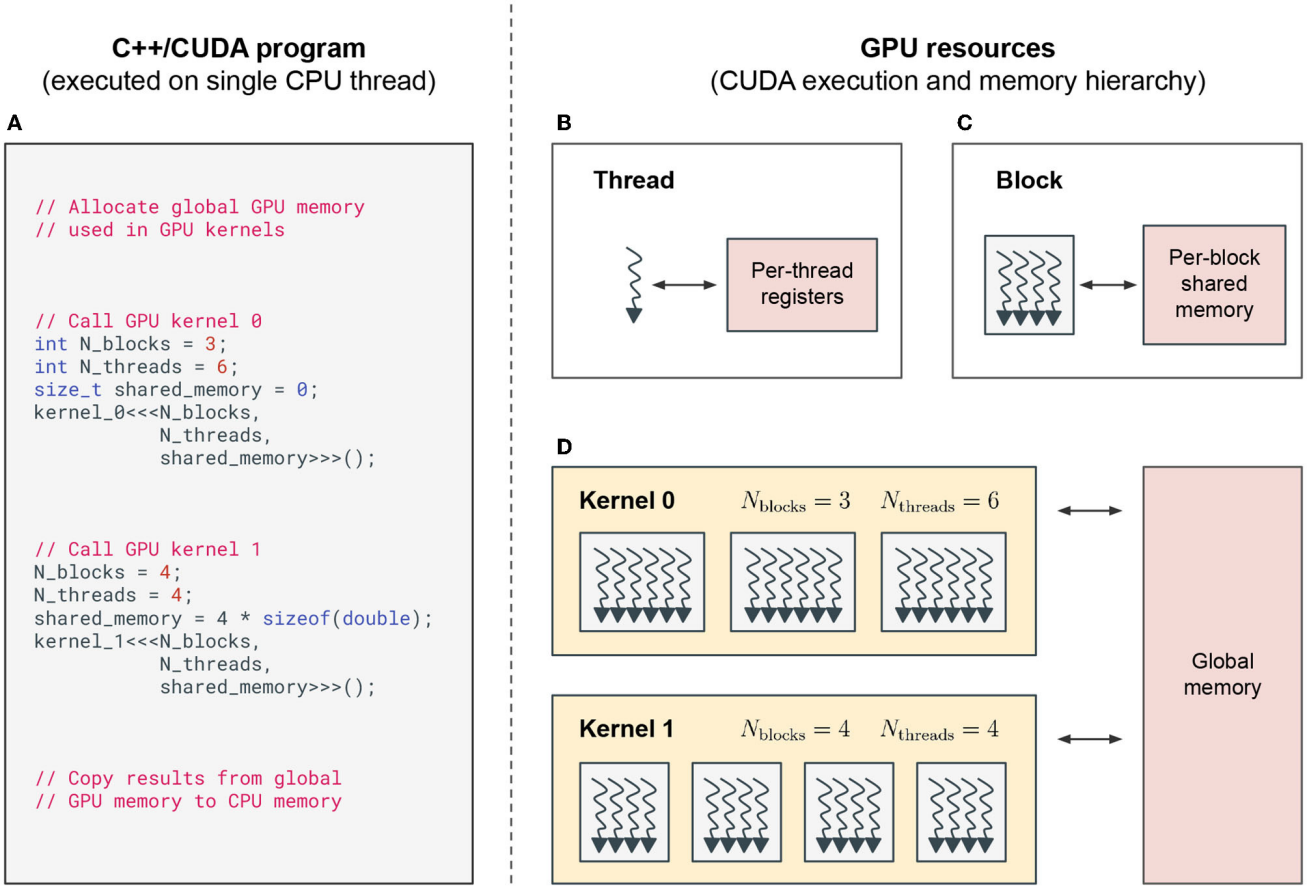
CUDA programming model. **(A)** A simplified, exemplary C++/CUDA program that is executed on a single CPU thread. The CPU manages memory on the GPU and calls CUDA kernels that are executed on the GPU. **(B–D)** GPU resources and CUDA execution and memory hierarchy when running CUDA kernels on the GPU. **(B)** Each CUDA thread on a GPU has access to its own memory registers. **(C)** Each CUDA block groups together multiple CUDA threads. All threads of the same block have access to the same shared memory. **(D)** CUDA kernels can be executed with different numbers of blocks *N*_blocks_, threads per block *N*_threads_ and shared memory per block. The kernels called in (A) are executed sequentially on the GPU, while multiple CUDA blocks are executed in parallel on the streaming multiprocessors (SMs) of the GPU. The example program in (A) calls kernel 0 without any shared memory and kernel 1 with enough shared memory to store one floating point number per thread. This memory could e.g., be used to calculate the sum of a variable over all threads in a block, using fast shared memory instead of slow global memory.

A typical C++/CUDA program is executed on a single CPU thread, which calls special GPU functions that are executed on the GPU (see Figure 2A). These functions are called CUDA *kernels*. When called, kernels execute their code in parallel by multiple CUDA *threads* (see Figure 2B), which are grouped into CUDA *blocks* (see Figure 2C). The number of threads per block *N*_threads_ and the number of blocks *N*_blocks_ in this thread hierarchy is set when calling the kernel (see Figures 2A,D).

##### 2.2.1.2. Memory Hierarchy

Each GPU has its own memory, which is separate from the CPU's memory. GPU memory is split into different types, which are hierarchically organized (see Figures 2B–D). *Global memory* is large (several gigabytes depending on specific hardware) and accessible by all threads, but memory access is very slow. *Shared memory* is accessible by all threads within the same thread block. This memory is much faster to access, but limited in size (up to a few megabytes split across all blocks). And finally, each thread has its own *registers* with the fastest access time, but which are also limited in number (up to a few megabytes split across all threads). Threads use registers to store the intermediate results during their computations, shared memory to communicate intermediate results during kernel execution between threads of the same block and global memory to communicate between threads in different blocks and to store results between kernel calls.

#### 2.2.2. Execution Control Logic

On the hardware level, NVIDIA GPUs consist of multiple streaming multiprocessors (SMs). During the execution of CUDA kernels, thread blocks are assigned to streaming multiprocessors (SMs) (see Figure 2). Each SM can execute a limited number of blocks concurrently, which are referred to as *active* blocks. All remaining thread blocks are queued for execution on the next available slot on any of the SMs. The maximal number of active blocks per SM depends on the resource requirements of the executed kernel and resource limits per SM (e.g., how many registers are required vs. available). When a block is executed on an SM, its threads are executed in groups of 32 threads, which are called *warps*. Each thread of a warp executes the same instructions at each clock cycle, which implements the single instruction multiple threads (SIMT) paradigm.

#### 2.2.3. Performance Considerations

##### 2.2.3.1. Occupancy

*Occupancy* per SM is defined as the ratio of active warps on an SM to the maximum number of active warps supported by the SM. Given the number of threads and blocks of a kernel and its resource requirements, an upper occupancy limit can be determined, the *theoretical occupancy*. There are multiple hardware limits that determine how well a kernel can be parallelized on the GPU. Here, we will only introduce a few of them which are relevant for our algorithms. Each SM has a limit on the number of threads in all active blocks, a limit on registers available to all threads in all active blocks, and a general limit on the number of active blocks. If any of these limits is exceeded, the number of active blocks per SM is automatically reduced such that the limits are fulfilled, reducing the theoretical occupancy of the kernel. Table 1 lists these limits for the GPUs used in this work.

**Table 1:** Hardware limits relevant to determine theoretical occupancy of GPU kernels.

**GPU (cc)**	**Active threads**	**Threads**	**Active blocks**	**Registers**	**Registers per thread**
	**per SM**	**per block**	**per SM**	**per SM**	**for 100% occupancy**
A100 (8.0)	2,048	1,024	32	65,536	32
RTX2080 Ti (7.5)	1,024	1,024	16	65,536	64

*The values are the same for all GPUs with the same compute capability (cc) and represent upper limits. To get the maximal registers per thread for 100 % occupancy, we divided the maximal registers per SM by the maximally active threads per SM. Only GPUs used in our experiments are listed*.

##### 2.2.3.2. Coalesced Memory Access

Memory accesses are issued in warps or half-warps (depending on the GPU and the memory request). When accessing global memory, always chunks of 32, 64 or 128 bytes are transferred – even if less memory was requested. That means, if a single thread wants to read 4 byte from global memory, a 32 byte transfer will be issued (the smallest transfer possible). If multiple threads read 4 byte from different non-contiguous memory addresses in global memory, there will be one 32 byte transfer per thread. But if all threads in a warp request 4 byte memory from contiguous memory addresses, a single 32 × 4byte = 128byte transfer will be issued to transfer all memory requested by all threads. This is called a *coalesced* memory access, which reduces latencies (i.e., waiting time) for global memory accesses significantly. Hence, it is crucial to layout data structures such that as many memory accesses as possible are coalesced.

### 2.3. Brian2CUDA Algorithms

Brian is a clock-driven simulator, which performs the same set of computations after each discrete time step Δ*t* of a simulation. In this section, we will explain the algorithms and data structures used in Brian2CUDA by going through the different simulation steps necessary to simulate one time step of the recurrent LIF network from Figure 1. All data structures introduced in the following reside in global GPU memory and all kernels introduced are executed sequentially on the GPU.

#### 2.3.1. Neurons

In Brian, neurons are defined in populations, where each neuron is described by the same set of dynamical equations and hence the same set of state variables (e.g., *V*_*i*_ in Figure 1B). In Brian2CUDA, for each neuronal population typically three separate CUDA kernels are defined: one for integrating the neuronal states (see Figure 1F), one for detecting spikes and one for resetting state variables of spiking neurons^2^. Since these computations are independent for all neurons, parallelization on the GPU is trivial: Each thread performs all computations for a single neuron. Nevertheless, it is important to coalesce global memory access (see Section 2.2.3.2). This is ensured in the integration kernel by storing neuronal state variables in contiguous global memory arrays (one entry per neuron) and accessing those such that consecutive threads access consecutive entries of the state variable arrays. In the spike detection kernel, the threshold crossing (typically of the membrane voltage) can be detected efficiently in parallel in the same way as in the integration kernel. The challenge here is to select and count the spiking neurons of the current timestep which naturally involves serialization. The implemented solution relies on a method from the CUDA programming API: threads that detect a spike perform an atomic increment of a population spike counter and use the counter value to store their neuron ID in a spiking neuron array. An atomic operation is an operation on a single variable that can be safely called by multiple threads, guaranteeing that all updates get applied correctly. These atomic increments limit the parallelization in the spike detection kernel when multiple threads try to increment the counter at the same time, and the writing of spiking neuron IDs into the spiking neuron array is generally not coalesced. The reset kernel is parallelized over spiking neurons and therefore the reading of spiking neuron IDs is coalesced, but the reset updates of the neuronal state variables are generally not. Since for the majority of models in computational neuroscience, the number of spikes per time step is much lower than the number of neurons per population, the potentially inefficient computations in the spike detection and reset kernels often contribute only little to the total computation time^3^.

#### 2.3.2. Synapses

A population of synapses in Brian is defined between a pre- and a postsynaptic population of neurons (for the recurrent synapses defined in Figure 1D, pre- and postsynaptic populations are the same). The simulation of synapses can generally be separated into synapse generation, synaptic state updates, spike propagation and synaptic effect application. The synapse generation in Brian2CUDA is performed on the CPU, using the same algorithm as Brian's C++ backend and thereby supporting all of Brian's connection methods. Synaptic state updates in Brian can be *clock-driven* or *event-driven*. Clock-driven updates are performed at every time step and are implemented in Brian2CUDA in a separate kernel in the same way as neuronal state updates. Event-driven updates are performed only when the pre- or postsynaptic neuron of a synapse spikes. These are performed during the synaptic effect application of the corresponding spike. With spike propagation, we refer to the processing of synaptic delays, which can be either *homogeneous* (the same for all synapses) or *heterogeneous* (varying across synapses). With synaptic effect applications, we refer to the modifications of synaptic target variables based on spikes (e.g., the reduction of the postsynaptic voltage potential by *J* for each presynaptic spike in the model from Figure 1). In Brian, both the pre- and postsynaptic spikes can have synaptic effects on pre- and postsynaptic neurons and the synapse itself. In the following, we will illustrate how Brian2CUDA implements spike propagation and synaptic effect application for different delay types and for the case of presynaptic neurons that modify postsynaptic variables, but the algorithms generalize to all other synaptic effect types. For both, spike propagation and effect application, kernels are parallelized over synapses in Brian2CUDA.

##### 2.3.2.1. Connectivity Information

**Figure 3:**
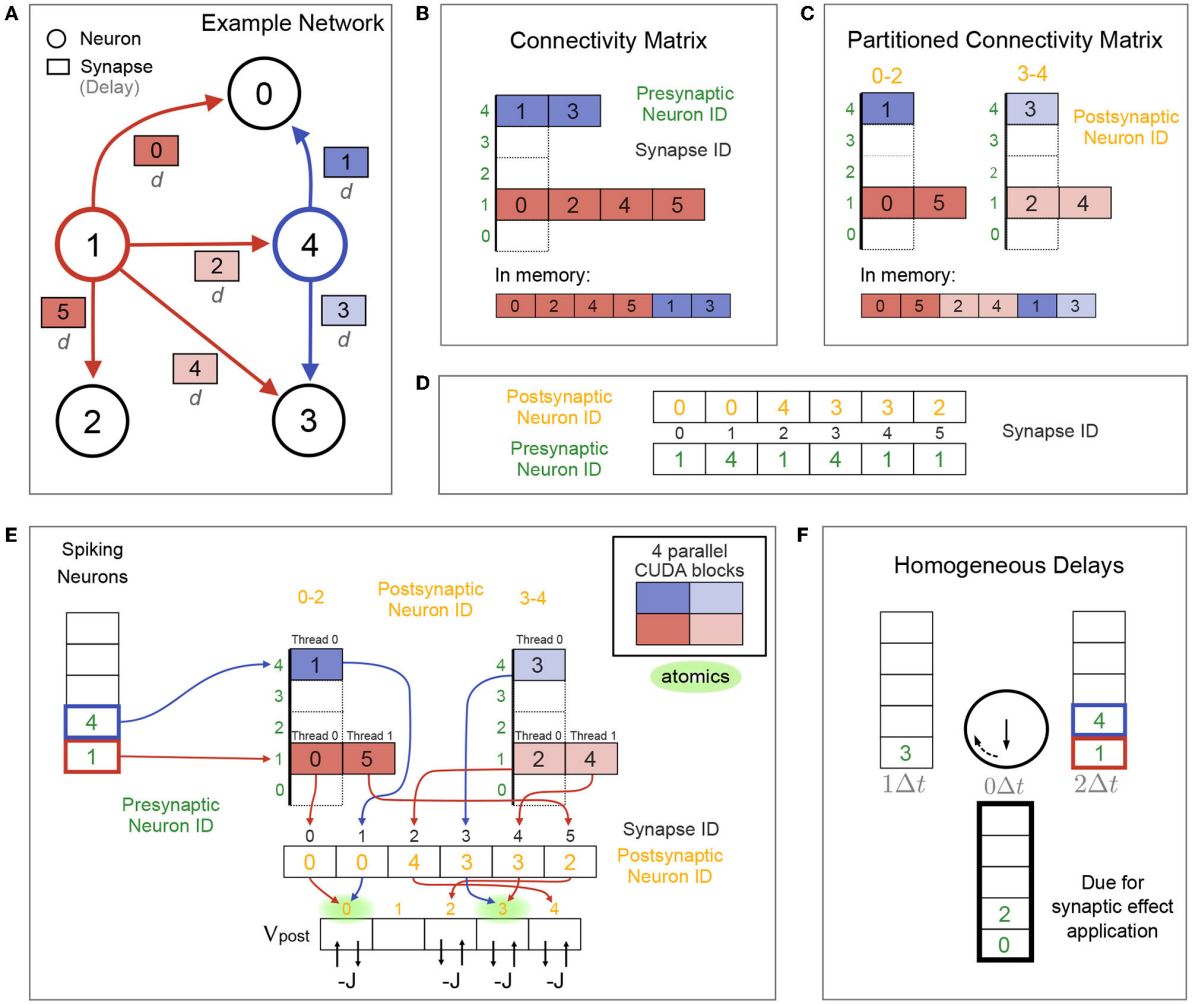
Synaptic algorithm for networks with no or homogeneous delays. **(A)** Example connectivity for the recurrent network from Figure 1, restricted to homogeneous synaptic transmission delays *d* and *N* = 5 neurons. Colored neurons 1 and 4 are spiking in the current time step. Color of their synapse IDs correspond to the parallelization over CUDA blocks in (E). **(B)** Connectivity stored in compressed form (YALE format) in global GPU memory as one concatenated array of synapse IDs sorted by presynaptic neuron ID (bottom view). Top view shows this array split by presynaptic neurons for visualization. Two additional arrays (not shown) store the start indices and number of synapses in the synapse array for each presynaptic neuron. Coloring correspond to the parallelization over CUDA blocks in (E). **(C)** Connectivity matrix for two postsynaptic neuron partitions, visualized as in (B). Each color shows one synapse group, defined by presynaptic neuron (red or blue) and postsynaptic partition (bright or dark). The synapse array is sorted in memory first by presynaptic neuron ID and then by partition (bottom view). **(D)** Pre- and postsynaptic neuron IDs for all synapses are stored in two arrays, sorted by synapses IDs. **(E)** Fully parallelized synaptic effect application for the network from (A) without delays (*d* = 0Δ*t*) and with the partitioned connectivity matrix from (C). Each of the 4 CUDA blocks (cf. colors) applies synaptic effects for all synapses of its respective synapse group. Membrane voltage updates are performed using CUDA's atomic operations to avoid race conditions. Potential atomic conflicts at the same memory location are marked in green. Without connectivity matrix partionioning (B), only two CUDA blocks (one per spiking neuron) would process the synapses (not shown). **(F)** Circular list of spiking neuron arrays for the network from (A) with homogeneous delays *d* = 2Δ*t*. Spiking neuron arrays are labeled with the time in which their synaptic effects are due for application. Spiking neurons of the current time step are stored in the array labeled with *d* = 2Δ*t*. Synaptic effects are applied for the neurons in the array labeled with 0Δ*t*. After each time step, all array labels are rotated clockwise and the applied spiking neuron array will be overwritten by the new spikes of the next time step.

Consider the example connectivity for our recurrent LIF network shown in Figure 3A, where synaptic effects are triggered by presynaptic spikes. The (sparse) connectivity matrix of synapse IDs sorted by presynaptic neuron ID is stored in YALE format (Figure 3B; Eisenstat et al. 1982). This connectivity matrix can optionally (via a Brian2CUDA preference) be split into multiple partitions of postsynaptic neurons, in which case synapses per presynaptic neuron are sorted by partition (Figure 3C). This creates *synapse groups* defined by presynaptic neuron and postsynaptic partition (different colors in Figure 3C). If synaptic effects are triggered by postsynaptic spikes, e.g., for models with spike-timing dependent plasticity (STDP), a separate connectivity matrix is created, sorted by postsynaptic neurons and partitioned by presynaptic neurons (not shown here). To access the neurons connected by a synapse, two additional arrays store the pre- and postsynaptic neuron IDs for all synapses, sorted by synapse ID (Figure 3D).

##### 2.3.2.2. Synapses Without Delays

When synapses have no transmission delay, there is no need for a separate spike propagation phase, and synaptic effects can be applied directly after spike detection. Effect application is parallelized over all synapses of all spiking neurons, where each CUDA block processes the synapses of one synapse group (Figure 3E). Each thread reads one synapse ID from the connectivity matrix, uses it as an index to read the postsynaptic neuron ID of that synapse, which is then used to index the postsynaptic membrane voltage. The synaptic effect (decreasing *V*_post_ by *J* in our inhibitory LIF network example) is performed using atomic operations to avoid race conditions from multiple threads writing to the same memory location, cf. Figure 3E. Reading synapse IDs from the connectivity matrix is coalesced, while reading the corresponding postsynaptic neuron IDs and membrane voltages is generally not. Partitioning the connectivity matrix can increase occupancy for networks with homogeneous delays if the overall number of spikes per time step is small enough (less than the limit on maximally active CUDA blocks; see Table 1) and the number of synapses per neuron is large enough (more than there are threads in each CUDA block). Under such conditions and without partitioning, there are too few active blocks with too many threads to parallelize all synaptic effects. Partitioning the connectivity matrix then moves threads from too full blocks into new active blocks, increasing parallelization.

##### 2.3.2.3. Synapses With Homogeneous Delays

When synapses have homogeneous delays *d* = *kΔt*, the spiking neuron array is stored for *k* time steps before the synaptic effects are applied. This results in a circular list of *k* + 1 spiking neuron arrays. Figure 3F shows an example for *k* = 2. The synaptic effect application algorithm is the same as for the no delay case (see Figure 3E), but using the neurons that spiked *k* time steps ago. Spike propagation for networks with homogeneous delays amounts to incrementing the circular list index referencing the spiking neuron array that is due for synaptic effect application. Therefore, adding homogeneous delays to a network comes at close to no computational cost at each time step, but increases memory requirements for storing multiple spiking neuron arrays.

##### 2.3.2.4. Synapses With Heterogeneous Delays

**Figure 4:**
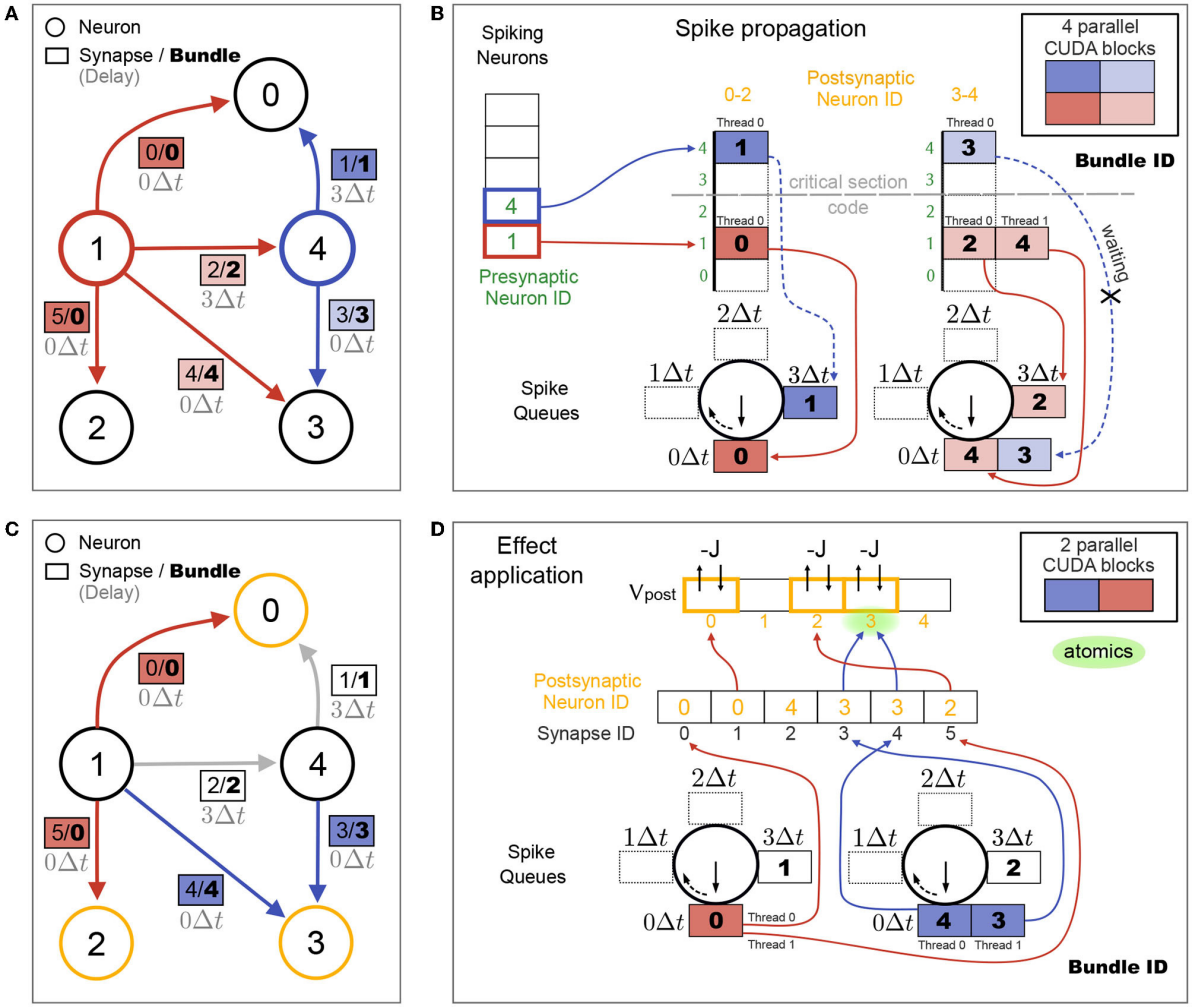
Spike propagation and synaptic effect application for synapses with heterogeneous delays. **(A)** Same connectivity as shown in Figure 3A, but with heterogeneous delays *d*_*ij*_ from neuron *j* to *i*. Neurons spiking in the current time step and their outgoing synapses are colored. Colors of synapse labels correspond to the parallelization over CUDA blocks during spike propagation in (B). Bundles group together synapses with same presynaptic neuron, postsynaptic partition as shown in (B) and delay value. All bundles for the same presynaptic neuron and postsynaptic partition define a bundle group (same color), each with a different delay. In this toy example, only bundle 0 has two synapses (0 and 5), the other bundles contain only one synapse. Additionally only the bright red bundle group consists of two bundles (2 and 4), while the other bundle groups contain only one bundle. **(B)** Spike propagation step. Bundles for all spiking neurons are sorted into spike queues based on their delay value and postsynaptic partition. Maximal delay in (A) is *d*_0,4_ = 3Δ*t*, requiring 4 spike queues per partition. Each of the 4 CUDA blocks propagates all bundles of its respective bundle group. A critical section code ensures that only one CUDA block per partition (red or blue) has access to the spike queues of its partition at any time. Two CUDA blocks of different partitions (dark and light) can operate concurrently on separate spike queues. For each partition, delay queues are constructed as a circular list of arrays and their labels are rotated at the end of each time step [after (D)] analogously to the circular list of spiking neuron arrays in the case of homogeneous delays in Figure 3F. **(C)** Synaptic effect application. Same connectivity as in (A). Colors now indicate neurons receiving synaptic effects in the current time step (yellow) and their incoming synapses (red and blue, these are the synapses from (A) without delay). Colors of synapse labels correspond to the parallelization over CUDA blocks during effect application in (D). **(D)** Effect application step. Synaptic effects of all synapses in all bundles in the 0Δ*t* spike queues are applied to their targets. One CUDA block per partition processes all bundles of its partition. Bundles are unpacked and each thread applies the effect on one synapse (e.g, two threads are processing the two synapses in bundle 0).

In networks with heterogeneous synaptic delays, synapses connected to spiking neurons are sorted into *spike queues* based on their synaptic delay. Analogously to the spiking neuron arrays used in the homogeneous delays case (cf. Figure 3F), *k* + 1 spike queues are created, which are arranged in a circular list and where *k* is the number of time steps in the highest delay in the network max(*d*_*ij*_) = *kΔt*. As before, the connectivity matrix can be partitioned by postsynaptic neurons, in which case each partition gets its own spike queues. To reduce the number of elements that need to be inserted into spike queues, the synapses with the same delay that would be propagated together, are grouped into *synapse bundles* and those bundles are inserted into the spike queues instead of synapses. Figure 4A shows the example network from Figure 3, but now with heterogeneous delays and additionally with synapse bundle IDs (instead of just synapse IDs). Figure 4B shows how the spike propagation algorithm sorts bundle IDs into spike queues. Since the maximal number of synapse bundles that will be stored in any of the spike queues is generally not known before a simulation, a custom dynamic vector implementation is used, which allows increasing spike queue sizes in GPU kernels on demand. This resizing requires reallocating spike queue contents in global GPU memory. While this is generally very expensive, it only happens at the beginning of a simulation until the spike queues are large enough and hence has an overall negligible effect on performance.

During spike propagation, parallelization is over synapse bundles, where each CUDA block operates on a different *bundle group* (different colors in Figure 4B; analogous to the synapse groups in Figure 3C). All CUDA blocks for the same postsynaptic partition and for different spiking neurons collect synapse bundles in the same spike queues. To avoid race conditions from potential memory reallocation, a critical section code allows only one CUDA block per partition to add bundle IDs into the spike queues at any time. All threads of this block can parallelize the pushing of synapse bundle IDs into the spike queues over threads, since each bundle with a different delay will be added to a different queue. Note that CUDA blocks from different postsynaptic partitions operate on different spike queues and can be executed concurrently. Therefore, increasing the number of partitions decreases the amount of serialization during spike propagation of heterogeneous delays. This can lead to better performance as long as the additional CUDA blocks don't exceed the maximal number of active CUDA blocks on the GPU (see Table 1).

During synaptic effect application, the synaptic effects of all synapses in the bundles in the 0Δ*t* spike queue are applied. In our toy example, where we only consider a single time step, these are all synapses without delays (Figure 4C). In general, multiple different synapses from neurons that spiked at different times are collected in each spike queue. Figure 4D shows how the synaptic effect application parallelizes over synapses. The number of CUDA blocks during effect application equals the number of partitions. A fixed number of CUDA threads per synapse bundle performs the effect application for all synapses of each bundle. In the present work, the largest bundle size is used as the number of threads per bundle, but this can be set by the user. Bundle sizes depend on the delay distribution and number of synapses per neuron in the network. If all bundles have the same size, each thread applies the synaptic effects of one synapse. The more bundle sizes vary, the less efficient is the parallelization given a fixed number of threads per bundle. In general, spike propagation performance benefits from partitioning the connectivity matrix as long as the typical size of the spike queue is larger than the number of threads per CUDA block.

### 2.4. CUDA Code Generation With Brian2GeNN

Our benchmarks compare Brian2CUDA's performance with the performance obtained when using the Brian2GeNN interface (Stimberg et al., 2020b). Since both are implemented as backends for the Brian 2 simulator, the exact same models can be run and easily compared. Note that the Brian2GeNN interface does not support synaptic connections with heterogeneous delays, therefore the corresponding benchmarks only compare Brian2CUDA to CPU performance.

The Brian2GeNN interface uses Brian's C++ framework to generate synaptic connections, initialize variables, and to generate the numerical update steps based on the given model equations. In the next step, the interface converts synaptic data structures and model descriptions to the GeNN format, and runs GeNN's own code generation process. Finally, the generated code gets integrated into a run loop running on the CPU that also takes care of exchanging memory between CPU and GPU when necessary (for details see Stimberg et al., 2020b). The internally used data structures and algorithms are identical to running a simulation with the GeNN simulator (for details see Yavuz et al., 2016). GeNN allows the user to choose data structures and algorithms most adapted to their model, and many of these choices are exposed in the Brian2GeNN interface. All benchmarks presented in this paper use GeNN's sparse connectivity method, and chose the—for the respective model configuration—faster of its two parallelization modes: *pre mode*, i.e., parallelization over pre-synaptic sources and sequential loops over post-synaptic targets, or *post mode*, i.e., parallelization over post-synaptic targets and sequential loops over pre-synaptic sources.

### 2.5. Benchmarks

#### 2.5.1. Benchmark Models

To assess the runtime performance of Brian2CUDA in comparison to Brian2 on CPU and Brian2GeNN we use as benchmarks different models that cover popular types used in computational neuroscience. Here, we give an overview of the model characteristics and behaviors. The simulation code with all model implementations, parameters and benchmark procedures that were used to generate the results of this paper are available in our Brian2CUDA GitHub repository^4^ and archived as Alevi et al. (2022).

##### 2.5.1.1. HH Benchmark: Hodgkin-Huxley Type Neurons With Static Synapses

For the first benchmark, we use a model of excitatory and inhibitory conductance-based Hodgkin-Huxley (HH) type neurons (also used in Brette et al. 2007; Stimberg et al. 2020b and based on Traub and Miles 1991). This neuron model consists of six coupled ordinary differential equations describing the dynamics of the membrane voltage, three gating variables, and excitatory and inhibitory synaptic conductances. We initialized membrane voltages and synaptic conductances independently from Gaussian distributions, such that all neurons had slightly different initial conditions (for details see Stimberg et al., 2020b). We simulated populations of *N* neurons (80% excitatory and 20% inhibitory) with random recurrent synapses. Synapses from spiking presynaptic excitatory and inhibitory neurons modify postsynaptic excitatory and inhibitory conductances based on their synaptic weights *w*_*E*_ and *w*_*I*_, respectively. Connectivity was randomly Bernoulli-sampled for each pair of neurons (including self-connections) with fixed probability $p=\frac{C}{N}$, where *C* = 1,000 is the average number of synapses per neuron. For *N* < 1,000, all neuron pairs were connected. The model is identical to the *COBAHH benchmark* in Stimberg et al. (2020b), where a mathematical description of the model and a list of parameters can be found.

**Figure 5:**
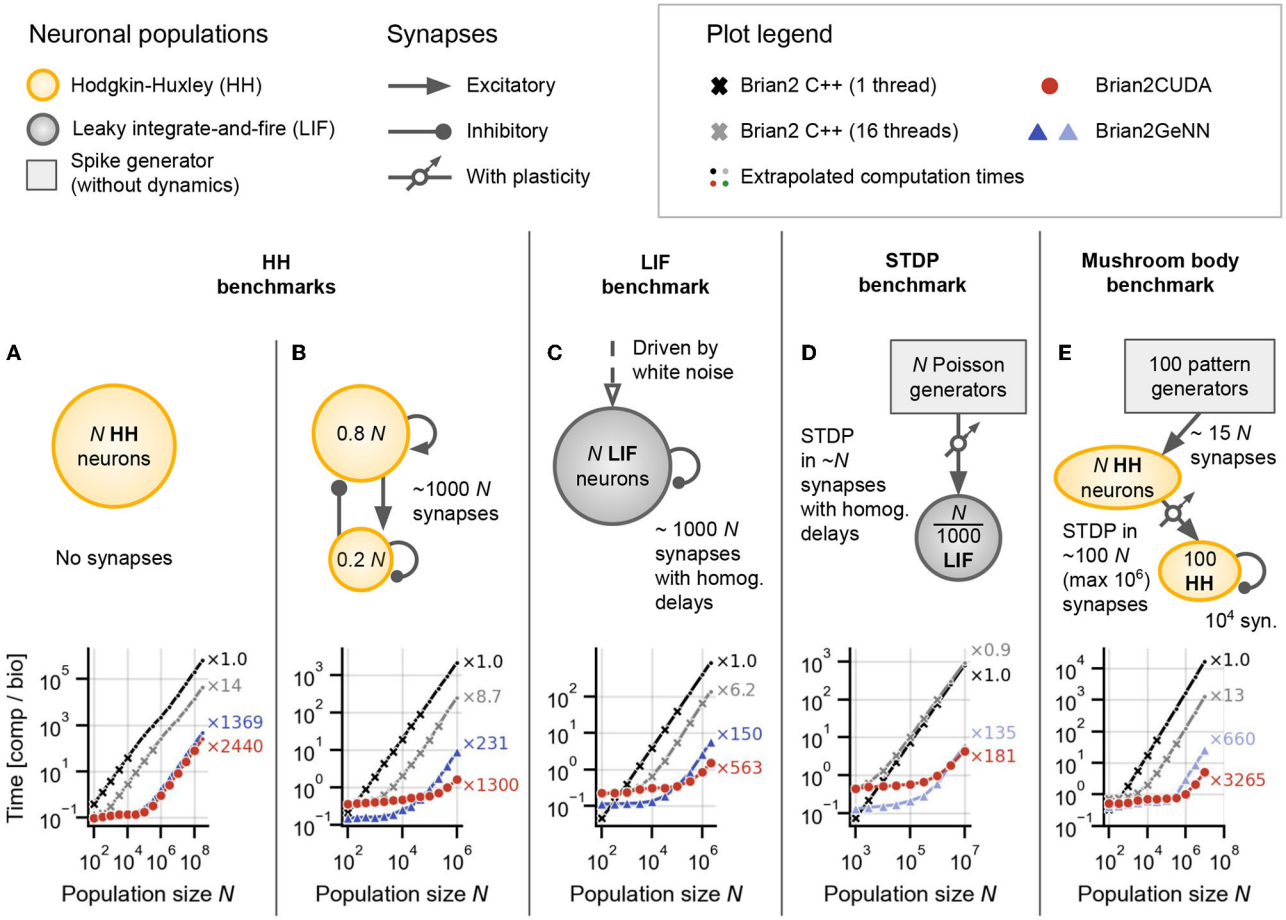
Benchmark results for networks without delays or with homogeneous delays. **(A)** Hodgkin-Huxley (HH) population without synapses. **(B)** Sparsely coupled recurrent HH network with 80% excitatory and 20% inhibitory neurons, without synaptic delays. **(C)** Leaky integrate-and-fire (LIF) network with sparse random connectivity and homogeneous synaptic delays *d*_*ij*_ ≡ *d* = 2ms for all synapses. **(D)** Spike-timing dependent plasticity (STDP) benchmark with homogeneous delays *d*_*ij*_ ≡ *d* = 2ms. **(E)** Mushroom body benchmark with non-plastic synapses in the first layer and synapses with STDP in the second layer, both randomly connected and without delays. For all panels: The text annotations on the right of the axes show the factor by which each simulation was faster than Brian's single-threaded C++ backend (i.e., obtained speedup) at the largest displayed *N*. Brian2CUDA was simulated without partitioning the connectivity matrix in all simulations (corresponding to Figure 3B). Brian2GeNN was simulated using its *post* parallelization strategy for **(A–C)** (dark blue) and *pre* parallelization strategy for **(D,E)** (light blue), which was the respective faster simulation mode compared to the other (not shown). All simulations were run once for 10s biological time. All times shown are computation times for the main time loop (i.e., without code generation, compilation, synapse generation or network initialization/finalization) and are relative to the simulated biological time. All simulations were interrupted if the main time loop took longer than 1,000s and the total computation time was extrapolated based on the fraction of biological time that was simulated (simulations for which this was done are indicated by small circular markers). All simulations were performed with Brian's single-precision preference for floating point numbers, i.e., 32-bit arithmetic, on A100 GPUs.

For Figure 5A, we simulated *N* neurons without any synapses, i.e., an uncoupled HH-type population. For an example of the activity in this network, see Supplementary Figure S2. For Figure 5B, we simulated the model with an average of *C* = 1,000 synapses per neuron and with random synaptic weights uniformly sampled from $w_{\text{E}},w_{\text{I}}\sim U\left(0,w_{\text{max}}\right)$ with $w_{\text{max}}=10^{-18}$S. The weights are chosen small enough to have no substantial effect on postsynaptic conductances such that the network activity does not change when increasing the population size, but synaptic propagation and effect application is still performed during the simulation (same procedure as in Stimberg et al., 2020b).

##### 2.5.1.2. LIF Benchmark: Noisy Integrate-and-Fire Neurons With Synaptic Transmission Delays

The LIF benchmark consists of a population of *N* noise-driven LIF neurons with recurrent inhibitory connections (based on Brunel and Hakim, 1999). This is the same model we introduced in Figure 1. The dynamics of each neuron are described by a single ordinary differential equation for the membrane voltage shown in Figure 1B. For all benchmark results, we simulated the model with spike threshold Θ = 20 mV, reset potential *V*_*r*_ = 10 mV, membrane time constant τ = 20 ms and inhibitory coupling *J* = 0.1 mV. Neurons have a refractory period of τ_ref_ = 2 ms. Recurrent random connectivity is implemented in the same way as in the HH benchmark, with connection probability $p=\frac{C}{N}$ with the same average number of synapses per neuron *C* = 1,000. Synapses from spiking presynaptic neurons modify postsynaptic membrane voltages.

**Figure 6:**
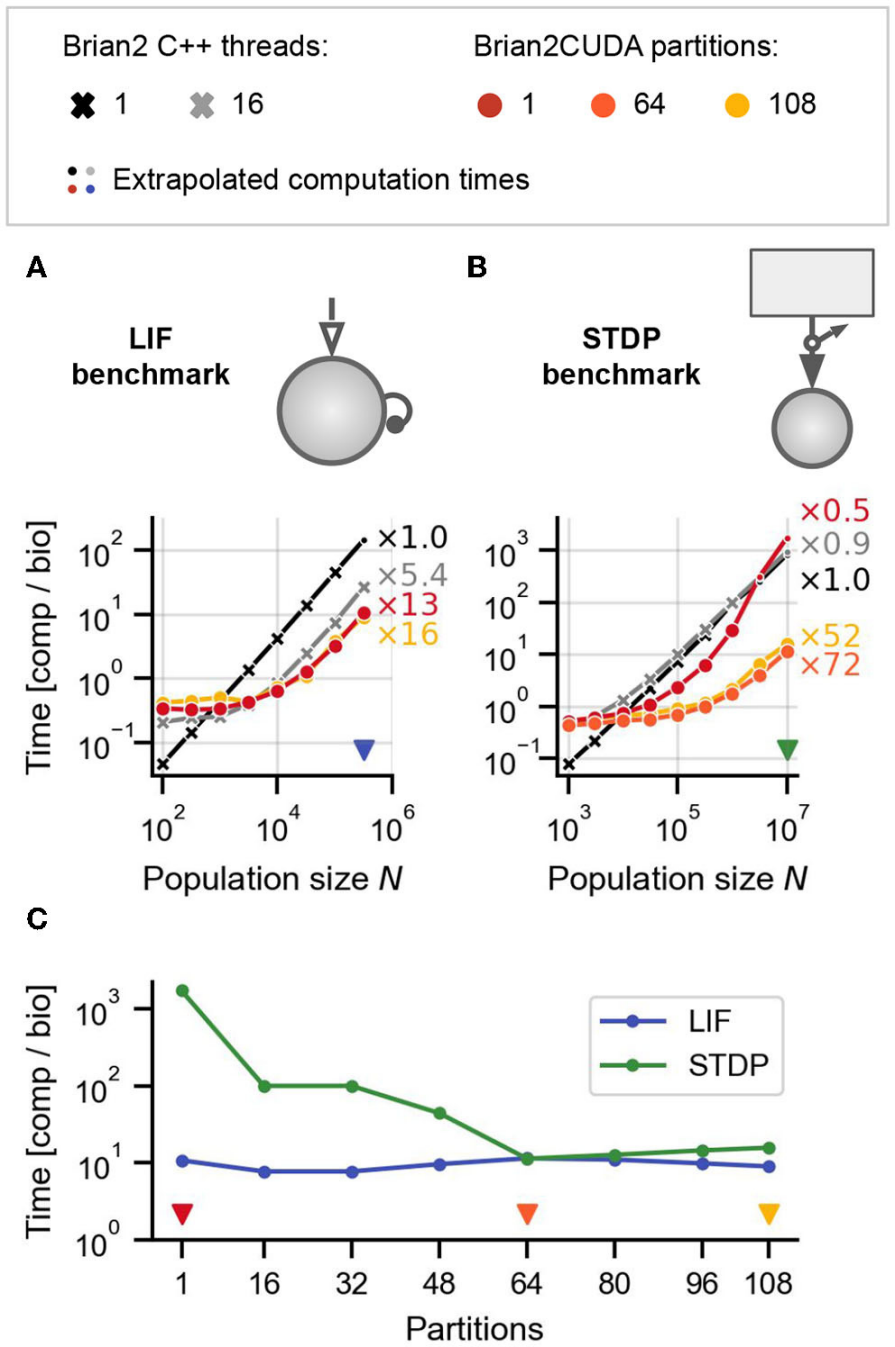
Benchmark results for networks with heterogeneous delays. **(A)** LIF benchmark model from Figure 5C but with heterogeneous delays. **(B)** STDP benchmark from Figure 5D but with heterogeneous delays. Delays for all synapses in both models **(A,B)** were uniformly sampled $d_{ij}\sim U\left(0,4\right)ms$. The external drive for the LIF benchmark was additionally modified to maintain the same regime of network activity as in the case of homogeneous delays (see Section 2.5.1.2). **(C)** Computation times for LIF (blue line) and STDP (green line) benchmarks for different numbers of connectivity matrix partitions in Brian2CUDA, for the maximal network sizes from **(A,B)** [indicated with blue and red triangular markers in **(A,B)**]. Triangular markers in **(C)** indicate the number of partitions plotted in **(A,B)** in the corresponding colors. All simulations were performed as described in Figure 5.

**Figure 7:**
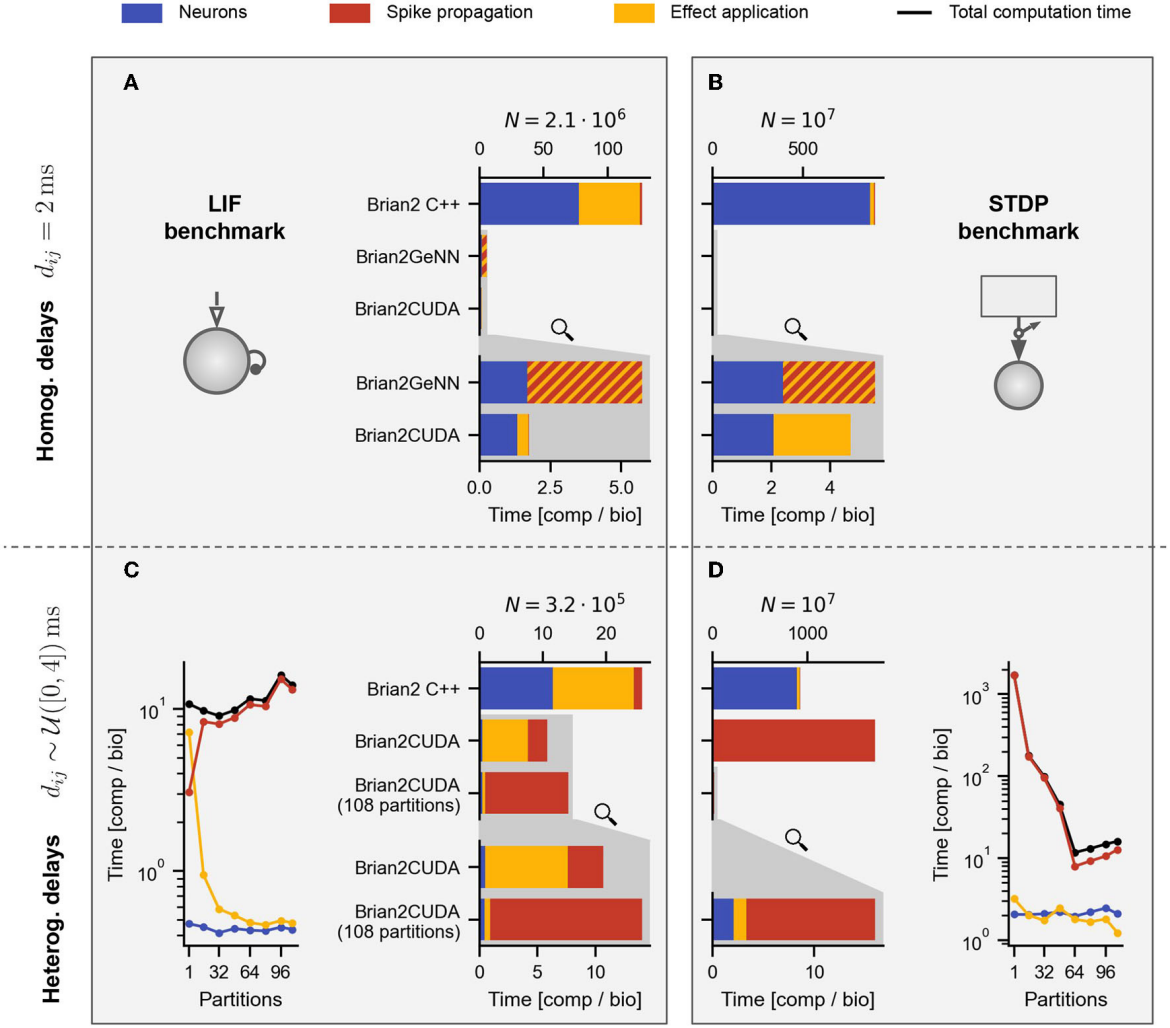
Profiling results for benchmarks with homogeneous and heterogeneous delays. **(A,B)** Profiling results for LIF **(A)** and STDP **(B)** benchmarks with homogeneous delays for the respectively largest population of Figures 5C,D. **(C,D)** Profiling results for LIF **(C)** and STDP **(D)** benchmarks with heterogeneous delays for the largest population size of Figures 6A,B. For the STDP benchmarks in **(B,D)**, the Poisson spike generators are included in the neuronal computation times (blue). The gray shaded areas in the lower part of **(A–D)** contain zooms of the respective GPU simulations in the middle (indicated by the magnifying glass symbol). (**C**, left) and (**D**, right) show profiling results for different numbers of partitions of the connectivity matrix in Brian2CUDA. Black lines are the total computation times for the main time loop (cf. Figure 6C). In all panels, Brian C++ was simulated with 16 threads, Brian2CUDA was simulated without connectivity matrix partitioning if not stated otherwise and Brian2GeNN was simulated in *post* mode for **(A)** and in *pre* mode for **(B)**. For Brian2GeNN, only the combined time of spike propagation and effect application (striped bars) was recorded since both are combined into a single CUDA kernel. All simulations were performed as described in Figure 5, but with enabled profiling measurements leading to slightly higher total computation times.

For the benchmark version with homogeneous delays (Figures 5C, 7A), the synaptic transmission delay was *d*_*ij*_ = 2 ms for each synapse from neuron *j* to neuron *i*. The parameters of the external drive (Gaussian white noise) were chosen as μ_ext_ = 25 mV and σ_ext_ = 1 mV. For the benchmark version with heterogeneous delays (Figures 6A, 7C), the synaptic transmission delays were uniformly sampled from a uniform distribution $d_{ij}\sim U\left(0,4\right)\text{ms}$. Resolved on the integration time grid with Δ*t* = 0.1 ms, this resulted in up to 41 different delay values. The external drive parameters were chosen as μ_ext_ = 27 mV and σ_ext_ = 0.33 mV. These parameters ensured that both benchmark versions had the same mean synaptic delay and that network activities showed qualitatively similar slow global oscillations (see Brunel and Hakim 1999; example activity for the heterogeneous version with *N* = 5,000 shown in Figure 1C).

##### 2.5.1.3. STDP Benchmark: Dynamic Synapses With Spike-Timing Dependent Plasticity

The spike-timing dependent plasticity (STDP) benchmark consists of *N* Poisson generators with dynamic feedforward synapses to a population of $\frac{N}{1,000}$ LIF neurons (for an example of the activity in the network, see Supplementary Figure S3). The Poisson generators have no dynamics that need to be integrated, but produce random Poisson spike trains with a mean firing rate of 15 s-1 (each generator performs one independent Bernoulli trial per time step). The dynamics of the LIF neurons are described by two differential equations, one for the membrane voltage and one for an excitatory synaptic conductance. The connection probability is $p=\frac{C}{N}$, where *C* = 1,000 is the average number of incoming synapses per LIF neuron (while each Poisson generator has on average only one outgoing synapse). Each synapse has a dynamic weight, which determines the synaptic effect that a presynaptic spike has on the postsynaptic neuron. The dynamics of the weights implement an all-to-all, additive STDP rule (Song et al., 2000; Morrison et al., 2008). Each pre- and postsynaptic spike increases a corresponding trace variable, stored for each synapse (necessary to support heterogeneous delays). In the absence of spikes, these trace variables decay exponentially, which is implemented through an event-driven update (see Section 2.3.2), and is therefore only calculated when necessary. When a presynaptic spike arrives, the current postsynaptic trace is used to decrease the synaptic weight, and conversely a postsynaptic spike triggers an increase in the synaptic weight based on the current presynaptic trace. Together, these changes implement the observed asymmetry of the STDP rule, where a presynaptic spike followed by a postsynaptic spike leads to synaptic facilitation, and the inverted sequence leads to synaptic depression (Bi and Poo, 2001). The technically challenging aspect of this model is that there are multiple synaptic effects triggered by pre- and postsynaptic spikes: synaptic trace variables are bidirectionally affected and additionally presynaptic spikes influence postsynaptic neurons *via* increasing the excitatory synaptic conductance. In Brian2CUDA, for this model, two separate connectivity matrices are generated, one for pre- and one for postsynaptically triggered synaptic effects. Both matrices are sorted differently, the former one by pre- and the latter one by postsynaptic neurons (see Section 2.3.2.1).

We use two versions of this model for benchmarking: with homogeneous delays (Figures 5D, 7B) and with heterogeneous delays (Figures 6B, 7D). The delays are the same as in the LIF benchmarks with the corresponding delay type. Note that the transmission delays are implemented as axonal delays, i.e., they only apply to synaptic effects triggered by the presynaptic population, while the synaptic effects from the postsynaptic population have no delays.

##### 2.5.1.4. Mushroom Body Benchmark: Complex Model With Multiple Neuronal Populations, Spike-Timing Dependent Plasticity and Noise

As the final benchmark (for Figure 5E), we consider a more “realistic,” complex model with multiple neuronal populations and synapse types, that combines several of the features of the previous benchmarks. For an example of the activity in the network, see Supplementary Figure S4. This model is inspired by the mushroom body of insects, based on the model by Nowotny et al. (2005), and used as a benchmark in earlier studies (Yavuz et al., 2016; Stimberg et al., 2020b). Briefly, this model consists of three populations: the first population consists of 100 pattern generators (i.e., does not simulate any dynamics but replays a pre-defined spike pattern), connecting to *N* HH-type neurons in the second population with a connection probability of *p* = 0.15 for each possible connection (Bernoulli sample). These connections are modeled as static, excitatory synapses. The neurons of the second population are modeled with the same equations (but different parameters) as in the HH benchmark presented earlier, except that they have no inhibitory conductance, which is not required without inhibitory synapses. This second population connects further to a third population of 100 HH-type neurons, with a connection probability of $p=\frac{10,000}{N}$ (with all-to-all connectivity for *N* < 10,000). These connections are plastic, following the STDP rule presented in the STDP benchmark. Finally, the third population has recurrent synapses to itself with all-to-all connectivity and static inhibitory synapses. For more details and parameters of this model, see Yavuz et al. (2016) and Stimberg et al. (2020b).

#### 2.5.2. Benchmark Procedure

All our benchmarks running on GPUs were executed on a single A100 data-center GPU (40 GB global memory), except for some results in **Figure 9**, which were executed on a single consumer-level GeForce RTX2080 Ti GPU (11 GB global memory). Brian's C++ backend was executed on an Intel Xeon Gold 6226R CPU with 16 physical cores, using 16 threads. Benchmarks were run on Brian2CUDA commit-tag paper2022^5^ (Alevi et al., 2022), Brian version 2.4.2 (Stimberg et al., 2020a), GeNN version 4.5.1 (Knight et al., 2021a) and Brian2GeNN commit 5f844d0 (based on version 1.6; Stimberg et al., 2021). We modified the Brian and Brian2GeNN versions with custom patches to execute our benchmarks and to get more detailed profiling information than available in the original implementations. Note that we ensured that these modifications had no significant impact on the runtime durations. The correct versions of these packages are stored as Git submodules in our GitHub repository, together with the necessary patch files and instructions on how to apply them. C++ code was compiled with gcc version 9.3.0 and CUDA code was compiled with nvcc version 11.2 based on CUDA toolkit version 11.2. The operating system on the computers with the A100 GPUs and Intel Xeon Gold 6226R CPUs was CentOS Linux release 7.4.1708 and on the computers with RTX2080 GPUs it was Ubuntu Linux version 20.04.3 LTS.

**Figure 8:**
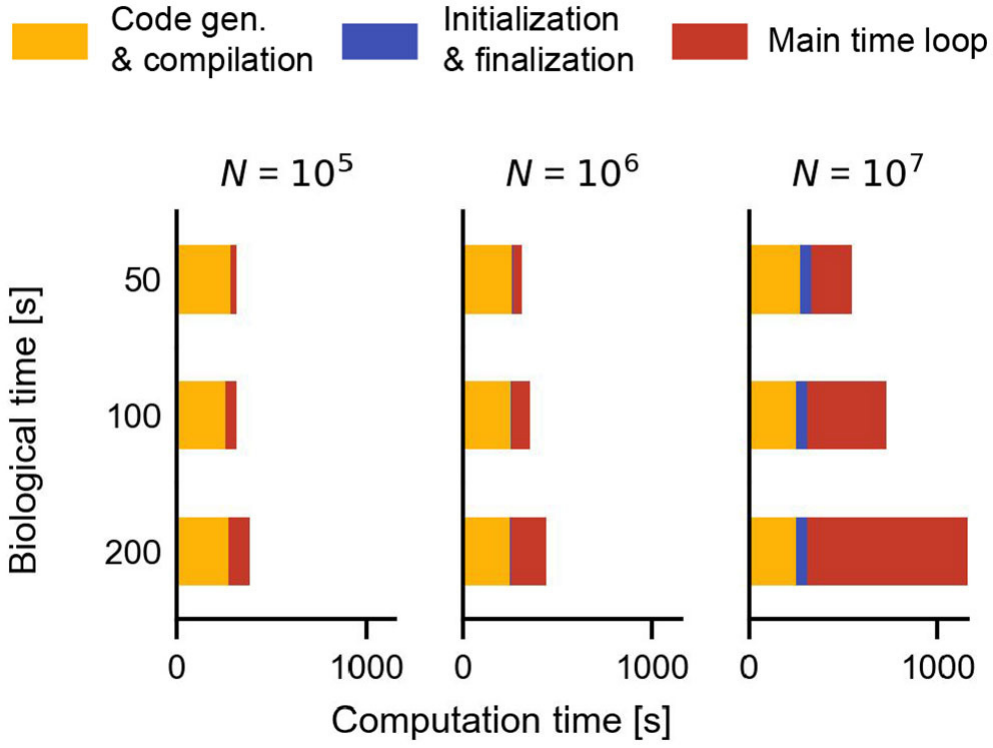
Additional time required during a simulation with Brian2CUDA for the STDP benchmark with homogeneous delays (Figure 5D). Code generation and compilation times (yellow) are independent of network size and biological time. Network initialization and finalization (blue) depend on network size but not on biological time. Simulation of the main time loop (red) scales with both, biological time (linearly) and with network size. Compilation was performed in parallel on 16 CPU threads.

For all benchmarks, we first recorded network activities for different network sizes and inspected that network activities were as expected. Additionally, we compared the results of Brian's C++ backend with the results of the Brian2CUDA backend for validation. For the final computation time measurements, we disabled the recording of any network activities. All benchmarks were simulated once for 10 s biological time (except for Figure 8) with a simulation time step of Δ*t* = 0.1 ms, and the computation times were divided by 10 to produce computation times relative to biological time (referred to as *Time [comp / bio]* in our figures). Simulations that exceeded 1,000 s of total computation time were interrupted before the end of the simulation (except for Figure 8). The computation time for the entire simulation was then linearly extrapolated based on the fraction of biological time that was simulated (data points marked in all figures). All figures except for Figure 8 show the computation time only for the main simulation loop, which consists of all simulation kernels that are executed at each time step of a simulation. This time does therefore not include compilation, network initialization, synapse generation, or result storage. For the Brian2CUDA profiling simulations in Figure 7, the CPU and GPU were synchronized after each kernel launch (forcing the CPU to wait for the kernel to terminate before continuing execution, which results in increased computation time) and kernel times were measured using timing functions in C++ code. For the Brian2GeNN profiling experiments, Brian2GeNN's own kernel timing preference was enabled, which records kernel times with CUDA events and without additional CPU/GPU synchronization. All Brian2CUDA simulations of benchmarks with no or homogeneous delays were executed without partitioning the connectivity matrix. For benchmarks with heterogeneous delays, the number of connectivity matrix partitions is shown in the figure labels or captions. In all benchmarks with heterogeneous delays, synapse bundles are used (and not individual synapses as Brian2CUDA can be configured to do). For Figure 8, the STDP benchmark with homogeneous delays was simulated for the biological times and network sizes shown in the figure legends and simulations were not interrupted after 1,000 s computation time. Code generation and compilation times were recorded from within the Brian package. To measure the initialization and finalization times, we computed the difference between the time spent within the main loop of the generated code and the total execution time of the compiled binary.

## 3. Results

To illustrate how different model features affect simulation performance on GPUs in Brian2CUDA and what speedup levels are typical, we consider multiple benchmark models covering various model types often used in computational neuroscience. In Sections 3.1–3.4, we focus on the computation time needed for the main simulation loop, which is the part of the simulation that is executed at every simulation time step. In particular, we summarize simulation performance for models without synaptic delays or with homogeneous delays in Section 3.1 and for models with heterogeneous delays in Section 3.2. In Section 3.3, we analyze the contributions of different algorithm parts to the runtime and in Section 3.4, we illustrate how recording of network activity and state variables influences runtimes. Section 3.5 then quantifies the overhead of preparing a simulation in terms of compilation and synapse initialization runtime beyond the main simulation loop. Finally, we show in Section 3.6 how the performance depends on the choice of floating point precision (single vs. double) and specific GPU hardware.

### 3.1. Benchmark Models Without Delays or With Homogeneous Synaptic Delays

#### 3.1.1. Hodgkin-Huxley Benchmark

To make efficient use of GPUs, simulation code has to perform highly parallel computations. The independent integration of neuronal state variables performed at each simulation time step for all neurons is trivial to parallelize on a GPU. In Brian2CUDA, each GPU thread computes the full state update for a single neuron (see Section 2.3.1). For a network of Hodgkin-Huxley (HH) type neurons without synapses, Brian2CUDA achieves a speedup of 3 orders of magnitude compared to Brian's single-threaded C++ backend for large enough network sizes (*N* > 10^5^; Figure 5A). The speedup of the GPU backend implemented in Brian2GeNN is comparable to the speedup of Brian2CUDA. With single-precision floats as shown here, Brian2CUDA performs slightly better than Brian2GeNN for large network sizes (Figure 5A), while for double-precision floats this difference is negligible (shown in Supplementary Figure S1F). Both backends also have comparable memory requirements, but Brian2GeNN is slightly more efficient. For example, on an RTX2080 Ti with 11 GB memory, Brian2GeNN can simulate a network that has about 1.4 times the size of the biggest network that can be simulated with Brian2CUDA (about 2.8·10^8^ vs. 2.0·10^8^ neurons).

Next we turn to networks with synapses, where the application of postsynaptic effects is less trivial to parallelize, since the effects of multiple spikes at the same target neuron cannot be applied at the same time in GPU memory (see Section 2.3.2.2). We therefore extend our benchmark model to a network of recurrently coupled HH-type neurons with conductance-based synapses without transmission delays (Figure 5B). In this model, each neuron has on average 1,000 synapses. To analyze the particular effect of the added recurrent synapses, we ensured that they do not change the network activity. In this benchmark, Brian2CUDA still achieves a speedup of 3 orders of magnitude compared to Brian's single-threaded C++ backend for large enough network sizes (*N* > 10^5^; Figure 5B). Notably, Brian2CUDA performs roughly 5 times faster than Brian2GeNN for the largest investigated network size (*N* = 10^6^), while being 2–3 times slower for smaller network sizes (*N* < 10^4^). The performance differences for small networks can be explained by the sequential execution from multiple small kernels in Brian2CUDA compared to the execution of fewer merged kernels in Brian2GeNN, see Section 4 for more details. In comparison to the network without synapses, the speedups gained through parallel computations in the multithreaded C++ backend and the GPU backends are reduced by a factor of 2–5 when including synapses (see Figures 5A vs. 5B). This illustrates that synaptic computations are generally less parallelizable than neuronal computations.

#### 3.1.2. Leaky Integrate-and-Fire Benchmark

The speedups of the GPU backends for the HH benchmarks demonstrate that neuronal computations benefit much more from parallelizations on the GPU than synaptic computations. Consequently, models for which the single-threaded C++ backend spends relatively less time for neuronal computations, should benefit less from computations on the GPU. To illustrate this effect, we next consider a population of noise-driven recurrently connected leaky integrate-and-fire (LIF) neurons with homogeneous synaptic transmission delays (based on Brunel and Hakim, 1999). This benchmark has the same number of synapses per neuron as the HH benchmark, but its neurons are described by only one dynamic state variable, compared to six state variables in the HH neuron model. Therefore, the single-threaded C++ backend spends relatively less of the overall computation time for neuronal computations when using LIF neurons. While Brian2CUDA still achieves a speedup of almost 3 orders of magnitude compared to Brian's single-threaded C++ backend for large enough network sizes (*N* > 10^5^; Figure 5C), the speedup is approximately halved compared to that of the recurrent HH benchmark (Figures 5B vs. 5C). Note that the addition of homogeneous synaptic transmission delays comes at almost no additional computational cost in Brian2CUDA (see Section 2.3.2.3). In relation to Brian2GeNN, Brian2CUDA performs 3 − 4 times better for the largest network sizes (*N* ≥ 10^6^), while being 2 − 3 times slower for smaller network sizes (*N* < 10^4^). As observed previously, Brian2GeNN is more memory-efficient than Brian2CUDA. It is able to simulate this benchmark on an RTX2080 Ti for a network with more than 2.0 · 10^6^ neurons, about 2.3 times the size supported by Brian2CUDA (about 8.6·10^5^ neurons).

#### 3.1.3. Spike-Timing Dependent Plasticity Benchmark

The benchmarks presented so far are based on static synapses, which do not change over the course of the simulation. However, an important subfield of computational neuroscience is interested in synaptic plasticity, where synaptic weights continuously adapt. Of particular interest in spiking neural networks are spike-timing dependent plasticity (STDP) rules, where the change in synaptic weight depends on the precise timing of pre- and post-synaptic spikes (Bi and Poo, 2001). Such plasticity rules present particular challenges for GPU acceleration, since they require more complex memory access patterns during the spike effect application phase than common static synapse models (cf. Brette and Goodman, 2012). To investigate the acceleration of models with STDP, we next examine a network with dynamic feedforward synapses from a large population of *N* Poisson generators to a much smaller population of $\frac{N}{1,000}$ LIF neurons (Figure 5D). The synapses in this network have (again) homogeneous transmission delays. Brian2CUDA achieves here 2 orders of magnitude speedup compared to Brian's single-threaded C++ backend for large enough network sizes (*N* > 10^6^), but the speedup is reduced by a factor of 3 compared to the LIF benchmark. This is due to the increased relative computation time required for synaptic computations from the STDP learning rule (as will be shown in more detail below). Compared to Brian2GeNN, Brian2CUDA is again slower for small network sizes (*N* ≤ 10^6^) while being slightly faster for the largest network size (*N* = 10^7^).

#### 3.1.4. Mushroom Body Benchmark

In the final benchmark on simulations without or with homogeneous delays, we consider a model of an insect mushroom body based on Nowotny et al. (2005), in an implementation already used in Stimberg et al. (2020b). It is a three-layer network with HH-type neurons and STDP in some of its synapses (Figure 5E). Since this model includes HH-type neurons with relatively few synapses, most of the computational effort is spent on the integration of the neuronal state variables. More precisely, there are two operational regimes: For smaller network sizes (*N* ≤ 10^4^), the number of synapses is 2 orders of magnitude higher than the number of neurons in the network and the performance is comparable to the HH benchmark with synapses (cf. Figure 5B). For larger network sizes (*N* ≥ 10^5^), the number of synapses is only 1 order of magnitude higher than the number of neurons and the performance is closer to that of the HH benchmark without synapses (cf. Figure 5A). Surprisingly, Brian2CUDA's speedup for large network sizes in the mushroom body model is even larger than for the HH benchmark without synapses. This behavior is probably due to an extra dynamic inhibitory conductance variable in the neuron model of the HH benchmark, which requires additional registers during the neuronal integration. Due to hardware limits of available registers on the GPU, this decreases the maximal theoretical occupancy of the neuronal integration kernel to 62.5 % for the HH benchmark compared to 100 % in the mushroom body benchmark (48 registers per thread vs. 32 registers per thread; cf. Section 2.2.3.1 and Table 1). Note that the number of registers needed by a kernel is not easily predictable, and does not directly reflect the number of state variables. The number of intermediate computation steps in the chosen integration method, additional temporary variables introduced by Brian's code generation process, but also the CUDA compute capability of the GPU and even seemingly irrelevant details such as the order of variable declarations, all affect register usage. This demonstrates that minor differences in a model can have large effects on performance.

Note that for Brian2GeNN, which performs about 5x slower for the largest network size, we again show the performance of its *pre* parallelization mode, for which the performance is better at larger network sizes. For smaller network sizes, Brian2GeNN in *post* parallelization mode performs slightly better (see Supplementary Figure S1E).

All results in Figure 5 are from simulations with single-precision floats and with preferences that gave the best performance. Results for additional preferences and simulations with double-precision floats are shown in Supplementary Figure S1.

### 3.2. Benchmark Models With Heterogeneous Synaptic Delays

Brian supports the simulation of networks with heterogeneously distributed synaptic delays. To simulate such networks, presynaptic spikes have to be sorted by delay and stored before their synaptic effects are applied. This spike propagation is challenging to parallelize efficiently on GPUs and additionally influences the parallelization of the synaptic effect application (Brette and Goodman 2012; Section 2.3.2.4). To evaluate the performance of Brian2CUDA's spike propagation and effect application algorithms, we include heterogeneously distributed synaptic delays in our LIF and STDP benchmarks, without qualitatively changing their network dynamics. We further evaluate Brian2CUDA's performance when partitioning its connectivity matrix (see Section 2.3.2.1).

Note that while the GeNN simulator has recently added support for heterogeneously distributed synaptic delays, this feature is currently not available in the Brian2GeNN interface. We therefore compare Brian2CUDA's performance only to Brian's C++ backends.

Figures 6A,B show the results for the LIF and STDP benchmarks, respectively. The performance of Brian's single-threaded C++ backend is not significantly affected by the presence of heterogeneous delays, while Brian2CUDA's performance drops by an order of magnitude for the LIF benchmark and between one and three orders of magnitude for the STDP benchmark (cf. Figures 5C,D), depending on the partitioning of the connectivity matrix. Note that Brian's multithreaded C++ backend does not efficiently parallelize spike propagation or the computations for Poisson generators, and hence performs similarly as its single-threaded backend in the STDP benchmark. Partitioning the connectivity matrix has little effect on overall runtime for the LIF benchmark, but increases performance by up to two orders of magnitude in the STDP benchmark. This strongly depends on the number of partitions and best performance was reached for 64 partitions (Figure 6C). To understand the effects of partitioning the connectivity matrix, we next consider profiling experiments to analyze the contribution of different parts of the simulation to the overall runtime.

### 3.3. Runtime Decomposition Into Different Algorithm Parts

We examine the contributions of the different algorithm parts by profiling the simulation. The following individual runtimes are available: the computation times for (1) performing neuron related computations (integration of dynamics and spike detection), (2) spike propagation and (3) synaptic effect application (including the event-driven integration of synaptic dynamics in the STDP benchmark). The decomposed runtimes for the LIF and STDP benchmarks with homogeneous delays are shown in Figures 7A,B, those for heterogeneous delays are contained in Figures 7C,D.

Brian's multithreaded C++ backend spends around half the computation time for spike propagation and synaptic effect application in the LIF benchmarks (Figures 7A,C yellow), while spending almost all time in the neuronal state updates and Poisson spike generation in the STDP benchmarks (Figures 7B,D blue). This is because the ratio of synapses to neurons (including Poisson generators) is much lower in the STDP benchmark compared to the LIF benchmark. The speedup of the GPU backends compared to the multithreaded C++ backend comes mostly from parallelizing the neuronal state updates and Poisson spike generation (including random number generation) on the GPU. When comparing Brian2CUDA and Brian2GeNN, both require similar times for the neuronal state updates and Poisson spike generation, but their efficiency for the synaptic effect applications differs for both benchmarks with homogeneous delays (Figures 7A,B). For the LIF benchmark, Brian2CUDA's synaptic effect application is more efficient compared to Brian2GeNN since the former parallelizes CUDA threads over all synapses while the latter parallelizes over postsynaptic neurons, requiring sequential looping over presynaptic spikes (using the *post* parallelization strategy of Brian2GeNN). In the STDP benchmark on the other hand, Brian2CUDA is only slightly more efficient in the synaptic effect application because Brian2GeNN's *pre* parallelization strategy is particularly suited to the case of many spiking neurons and few postsynaptic partners as explained above.

For heterogeneous delays, Brian2CUDA spends most of the computation time on spike propagation and synaptic effect application relative to neuronal state updates (Figures 7C,D). For the LIF benchmark with heterogeneous delays, increasing the number of partitions increases spike propagation times but decreases synaptic effect application times (Figure 7C). Each neuron in this benchmark has on average 1,000 synapses grouped into 41 synapse bundles per partition (see Section 2.3.2.4). Without partitioning the connectivity matrix, each CUDA block sorts all synapse bundles of one spiking neuron into spike queues, using one CUDA thread per bundle. This results in small CUDA blocks with only 41 active threads during spike propagation. For the large network size here, the number of spikes per time step is of the same order as the maximal number of active CUDA blocks on the GPU (see Table 1). Partitioning the connectivity matrix under these conditions reduces the size and increases the number of the already small CUDA blocks without being able to execute them concurrently. Consequently, spike propagation times increase with partition number (Figure 7C, red). On the other hand, the synaptic effect application profits from partitioning the connectivity matrix. Without partitioning, only a single CUDA block applies all synaptic effects of the spike queue for the current time step. For large networks with large spike queues, partitioning distributes synapses across multiple CUDA blocks, significantly increasing effect application performance (Figure 7C, yellow). Note that partitioning also has a small impact on the memory usage: on an RTX2080 Ti, Brian2CUDA can simulate a LIF network with heterogeneous synapses with around 5.6·10^5^ neurons when using a single partition, but with only about 0.77 times the size (around 4.3·10^5^ neurons) when using 68 partitions.

For the STDP benchmark with heterogeneous delays, increasing the number of partitions decreases both, the spike propagation and the effect application times up to an optimal number of 64 partitions (Figure 7D). Without partitioning, the spike propagation is so inefficient that the total runtime exceeds that of the single-threaded C++ simulation (cf. Figure 6B). For this benchmark, every Poisson neuron has on average only 1 synapse. This is the worst-case scenario for Brian2CUDA's spike propagation algorithm, since all CUDA blocks have only a single thread and the hardware limit on maximally active blocks per SM strongly limits the number of synapse bundles that can be added to the spike queues concurrently. Additionally, the resulting small workload per SM leads to a low parallelization across active CUDA blocks since only one CUDA block can access all spike queues at any time. Increasing the number of partitions also partitions the spike queues. Since concurrent access of different CUDA blocks to different spike queues is possible, this increases spike propagation performance. This benchmark shows that for large neuronal populations with extremely sparse synapses (here only 1 synapse per neuron), Brian2CUDA's connectivity matrix partitioning can have drastic benefits on performance.

### 3.4. Runtime Contribution of Network Activity and State Variable Recordings

To analyze a spiking network model, Brian allows recording spike times, state variables and population firing rates. In Brian2CUDA, recorded variables are stored in GPU memory during a simulation and are transferred to CPU memory and written to disk at the end of a simulation. The contribution to overall computation time of such recordings strongly depends on the details of a model (e.g., neuronal firing rates) and the number of recorded variables.

Consider for example Figure 1C, which shows the results for a simulation of the LIF benchmark with heterogeneous delays. To record this data, a spike recorder records all spikes in the network, a state variable recorder records the voltage of a single neuron for all time steps of the simulation and a population rate recorder records the fraction of spiking neurons at each time step. When adding these recorders to the largest LIF network with heterogeneous delays shown in Figure 6A (*N* = 3.2·10^5^), they require around 6 % of the computation time in the main simulation loop. Half of this additional time is spent on the spike recordings. For networks with overall less computation time per recorded unit, the contribution of recordings to total computation time naturally increases. For the extreme case of the HH benchmark without synapses (Figure 5A; recorded data shown in Supplementary Figure S2), the same recordings as above require around 40 % of the computation time for *N* = 10^6^ neurons. Of this additional time around 2/3 is spent on spike recordings.

While Brian2CUDA stores recordings in GPU memory until the end of a simulation, Brian2GeNN copies them at each time step from GPU to CPU memory. Therefore, GPU memory can be a limiting factor for recordings in Brian2CUDA, whereas Brian2GeNN requires very little GPU memory. For the HH benchmark above, spike and population rate recordings in Brian2GeNN perform similarly to those in Brian2CUDA, while state variable recordings perform significantly worse. This is because of an inefficient implementation of the state variable recorder in Brian2GeNN, which copies at each time step all state variables of all neurons to the CPU, independent of the number of recorded neurons. This results in up to 2 orders of magnitude longer computation times when recording a single voltage trace in the HH benchmark example above.

Compared to Brian's C++ backends, absolute network recording runtimes in Brian2CUDA are comparable for large recordings (e.g., for the HH benchmark example), and can be slower for smaller recordings (around 5 times slower for LIF benchmark example). This is because memory copies in GPU memory are slow, and Brian2CUDA benefits more from parallelizing the copy process for larger recordings. Given the large speedups for other computations in Brian2CUDA, network activity and state variable recordings contribute relatively much more to total computation times than in Brian's C++ backends.

### 3.5. Additional Computation Time Factors: Code Generation, Compilation, Initialization, and Finalization

So far we have been analyzing the computation time needed for the main simulation loop, i.e., only that part of the simulation that is executed at every simulation time step. For long running simulations of large networks or for real-time applications, this is the most relevant performance measure. But in order to get from a Brian model script to the results, the Python code needs to be translated into the target language, which needs to be compiled and executed and finally, the results need to be transferred back into the Python environment. Furthermore, at the beginning of the simulation, the model needs to be initialized, which includes generating synapses, setting up connectivity matrices in the necessary format and for GPU backends, transferring data to GPU memory. Figure 8 shows how compilation and network initialization contribute to the overall execution time for the STDP benchmark with homogeneous delays simulated with Brian2CUDA (cf. Figure 5D). The time spent in the simulation loop is proportional to the simulated biological time and also depends on the population size *N* of the network. The initialization time during the simulation is independent of the simulated biological time and increases with network size. And finally, the compilation time is independent of both, the simulated biological time and the network size.

Generally, for smaller networks (here *N* ≤ 10^6^) with shorter biological times (here *T* < 200 s), most of the computation time is spent on compilation, while this becomes negligible for larger networks simulating longer biological times. The compilation time for Brian's C++ backends Brian2GeNN and Brian2CUDA is mostly comparable, but can differ for some models. Brian's C++ backends and Brian2CUDA generate separate (CUDA C++) source files for each neuronal population object or synapse object. This can increase compilation times for networks with many objects, in particular for Brian2CUDA, which suffers from slower CUDA code compilation. The mushroom body benchmark for example requires almost twice the time for compilation as the STDP benchmark shown here because it consists of twice as many neuronal populations and synapse groups. While Brian2GeNN requires additional time for first generating GeNN code from the Brian model, it can compile the final CUDA code faster because it merges multiple computations into fewer CUDA kernels and source files.

### 3.6. Dependence on Floating Point Number Precision and GPU Hardware Choice

**Figure 9:**
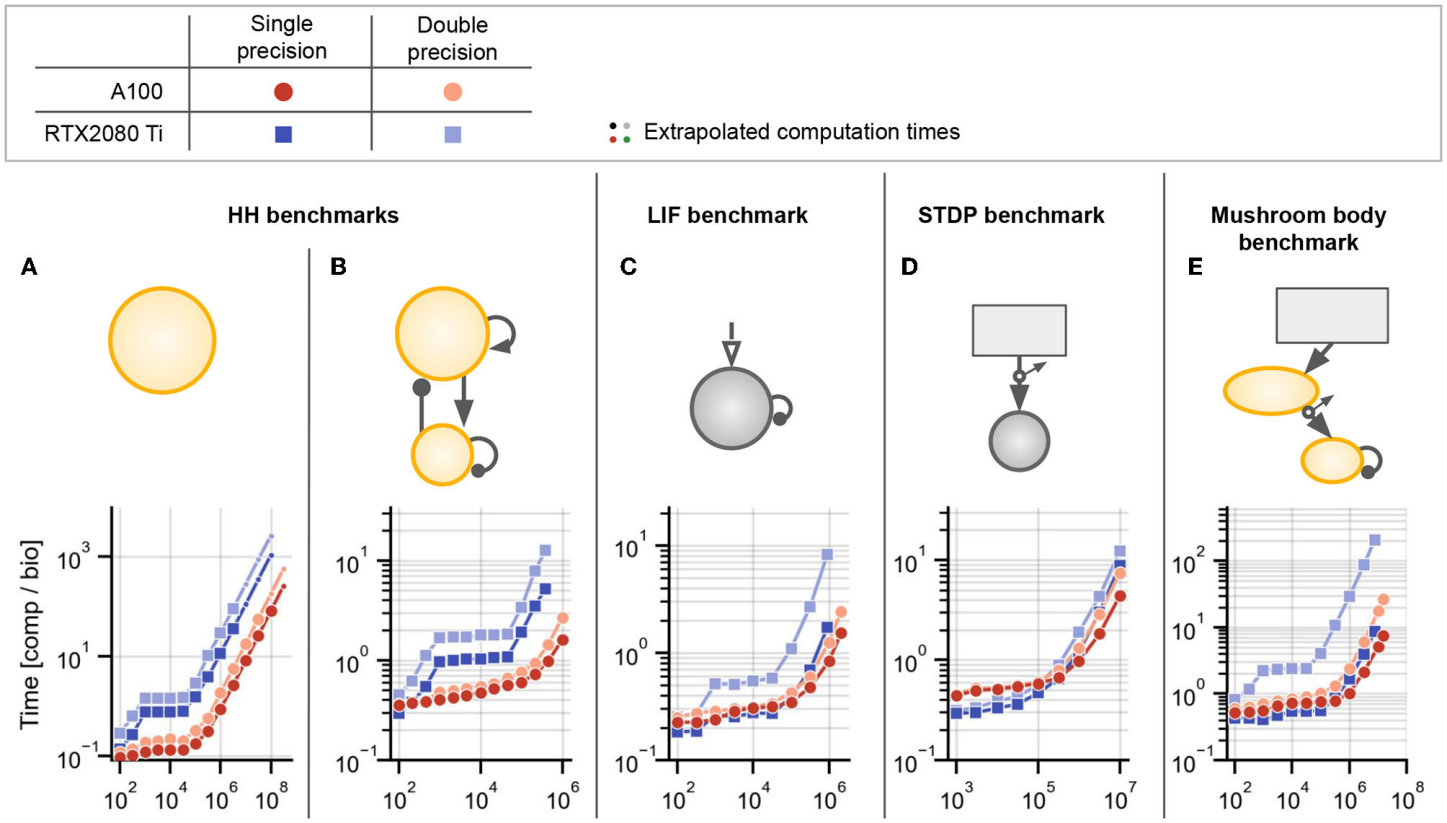
Benchmark results for single- vs. double precision on consumer-grade vs. data-center GPUs. **(A–E)** Same benchmarks as shown in Figure 5. Simulated without connectivity matrix partitioning in Brian2CUDA, with single-precision floats (dark colors) and double-precision floats (bright colors) and on A100 data-center GPUs (red colors) and GeForce RTX2080 Ti consumer-grade GPUs (blue colors). Dark red lines show same data as in Figure 5.

The results above stem from simulations using single-precision floating point arithmetics on A100 data center GPUs. Here we compare those results with Brian2CUDA's performance for simulations with double-precision floats on A100 GPUs and using a more affordable hardware GeForce RTX2080 Ti consumer-grade GPU (Figure 9). Consumer-grade GPUs are typically optimized for single-precision arithmetic operations and often have very low processing power on double-precision floats. The processing power of the RTX2080 Ti for double-precision floats is ~32 times lower than for single-precision floats, while for the A100 it is only ~2 times lower. The processing power of single-precision floats on the A100 is ~1.66 times larger than on the RTX2080 Ti. However, the performance differences between GPUs and between single-precision and double-precision simulations don't necessarily reflect the difference in processing power. Additional factors play a role, such as hardware limits on memory per SM or available data transfer bandwidths. Specifically, the hardware limits can have double effect when comparing single- to double precision simulations. For the mushroom body benchmark, the speedup from double- to single-precision simulations on the RTX2080 Ti (Figure 9E, bright blue vs. dark blue) is much higher than in the other benchmarks. This is not only because of the increased processing power, but also because for double-precision simulation the extended memory requirements reach the hardware limits on available registers (see Table 1), forcing the simulation to run with less active threads. With single-precision floats, the reduced memory requirements allow higher GPU occupancy on top of the higher processing power for single-precision floats. Additionally, only computation bound simulations will show strong performance differences between floating point precisions and GPUs (e.g., in the HH benchmarks; Figures 9A,B). For simulations which are bound by communication tasks such as spike propagation, the performance differences are much lower (e.g., in the STDP benchmark; Figure 9D).

In summary, these results show that one does not need extremely expensive data-center GPUs to benefit from GPU computations in spiking neural networks, since much cheaper consumer-grade GPUs can perform comparably for many model types—at least for simulations with single-precision floats.

## 4. Discussion

Building on the user-friendly simulator Brian and its code generation framework, the Brian2CUDA package presented here allows users with little technical expertise to simulate arbitrary neural and synaptic models on GPUs. As we have shown, this can lead to an important acceleration of a wide range of model simulations. The achievable speedup depends on the details of the model and the size of the network. For a small network, or a model with challenging features for parallelization such as heterogeneous transmission delays, only a several-fold increase in simulation speed might be possible. On the other hand, for models that are more favorable to parallelization, such as unconnected networks or networks with homogeneous delays and complex neuron models, the simulation speed can increase dramatically by several orders of magnitude. Our detailed benchmarking has shown a number of possible routes to further optimize the simulation speed for the challenging situations, which we will discuss in the following section.

### 4.1. Limitations and Future Work

Efficiently simulating different types of synaptic models on GPUs is challenging because there is no single algorithm that is best for all situations (Brette and Goodman, 2012; Kasap and van Opstal, 2018). Through partitioning the connectivity matrix, Brian2CUDA can counteract performance degradation for some cases where the default parallelization strategy would be inefficient. For models with homogeneous transmission delays and without partitioning the connectivity matrix, the effect application of individual spiking neurons is parallelized over CUDA blocks. Partitioning the connectivity matrix distributes the synapses for each spiking neuron over additional CUDA blocks. However, this increases performance only when the number of synapses per spiking neuron is larger than the maximal number of threads per CUDA block (1,024; cf. Table 1). For all benchmark models here, the average number of synapses per neuron is ≤1,000, for which partitioning does not increase parallelization. For models with more synapses, however, partitioning is expected to be beneficial as long as the number of spiking neurons per time step is small enough in order to keep the total number of CUDA blocks below the hardware limit on active blocks on all SMs of a GPU. For models with heterogeneous delays, partitioning the connectivity matrix has a non-trivial effect on spike propagation and synaptic effect application algorithms (see Figure 7). For example, without partitioning, spike propagation is very efficient while effect application is inefficient due to only one CUDA block applying all synaptic effects. Future work can further accelerate the simulation of models with heterogeneous delays by parallelizing the effect application over more CUDA blocks instead of using only one CUDA block per partition.

The current implementation of Brian2CUDA is optimized for large networks, where its speedups compared to Brian's C++ backends are the largest and where it outperforms Brian2GeNN in simulating the benchmark models employed here. For smaller network sizes, however, Brian2CUDA is often outperformed by Brian2GeNN (see for example Figures 5B–D). This can be at least partly explained by Brian's modular approach, inherited by Brian2CUDA. Each individual model component—e.g., the numerical integration, the thresholding, the resetting (cf. Section 2.3)—is contained in an individual kernel, and all kernels are executed sequentially. For kernels that don't utilize all resources (e.g., small populations of synapses/neurons), this leads to performance degradation. In contrast, Brian2GeNN merges all calculations related to neurons into one kernel and all updates of synapses in another kernel. We are currently working on two features that are promising to increase performance for smaller networks: (1) Using CUDA's concurrent kernel execution capabilities, kernels for separate neuron and synapse objects can be executed in parallel while keeping Brian's modular approach. (2) Convenience functions in Brian's Python interface can be implemented that allow users to easily merge multiple versions of the same (potentially small) model into a single large model. This would not only allow much easier parameter explorations of networks on a single GPU but also benefit from Brian2CUDA's optimizations for large networks.

Brian2CUDA's main focus is on optimizing the simulation phase, since this typically dominates the overall time for larger networks. To run smaller networks or simpler simulations, however, the long code generation and compilation phase in the beginning (cf. Figure 8), can be a major inconvenience. The long compilation times partly stem from Brian's modular approach mentioned above. Each component of the simulation is contained in a separate code file that needs to be compiled individually. To reduce compile times, multiple code files could be combined during code generation. It should be noted, however, that the reported compilation times are the full compilation times for a new simulation. If a user re-runs an existing simulation and only changes some aspects of it, only the changed source code will be re-compiled.

Another major future optimization for network simulations that run for a short biological time, is the synapse generation in the initialization phase of the simulation. At the moment, synapse generation uses Brian's C++ mechanism and therefore does not benefit from the GPU at all.

Current data structures and algorithms for simulating synapses are designed to handle all synaptic models and connection structures supported by Brian. But they perform better on some model types than on others. For example, for homogeneous delays, our synaptic effect application algorithm performs best when the number of connections is equally distributed across neurons. For structured connectivity, variable synapse group sizes can lead to unbalanced workloads across CUDA blocks during effect application (cf. Figure 3E), which can affect performance. Similarly, for heterogeneous delays, our synaptic effect application algorithm for synapse bundles performs best when bundle sizes are uniformly distributed within each synapse group because the same number of threads is assigned to each bundle (cf. Figure 4D). Strong variability across bundle sizes would lead to unbalanced workloads across groups of threads processing synapses of different bundles. In order to avoid these unbalanced workloads, one future direction could be to optimize our connectivity matrix scheme based on connectivity details. This could allow distributing workloads more evenly or exploiting local connectivity structures in our algorithms (as has been done before by Fidjeland et al., 2009; Fidjeland and Shanahan, 2010).

The presented work mostly focussed on optimizing simulation performance and less on memory usage. However, available memory can be a major constraint, in particular on consumer-grade GPUs. This is especially true when recording spike times and state variables from many neurons or synapses, which Brian2CUDA stores in GPU memory (see Section 3.4). In future versions, we plan a recorder implementation that allows transferring data in regular intervals from GPU to CPU memory, and we will focus on optimizing further unnecessary redundancies and memory inefficiencies. This should close the gap to Brian2GeNN, which is currently more memory-efficient.

Brian2CUDA is designed to support all features of Brian2 that are currently supported by its C++ backend and builds on the same code generation framework. It is therefore considerably less limited than the Brian2GeNN backend (discussed below), and supports a large variety of models. We have focused the development on spiking networks of single-compartment models, since they are most likely to benefit from GPU acceleration. Nevertheless, Brian2CUDA has support for other types of models supported by Brian, such as multi-compartment models, or rate-based models. This support is preliminary, though, and using Brian2CUDA might not give any performance benefits prior to improving the respective parallel algorithms.

As a general note on the limitations above, we would like to again emphasize that due to Brian2CUDA's implementation as a backend for the Brian simulator, a researcher does not need to invest any additional time or effort to port a model to Brian2CUDA. In contrast, porting a model to a simulator that only targets the GPU carries the risk that the effort is not worth the benefit. Due to the backend approach, researchers can also easily switch between the CPU and GPU-based approaches during development of a new model. For example, a researcher can do the initial development and testing on a small-scale model with the CPU, without having to pay the additional cost for the CUDA compilation, and then switch to the GPU for the final model, where the slower compilation is more than compensated by the faster computation time.

The Brian2CUDA backend is currently only supported for Linux operating systems (in contrast to Brian which supports Windows, Linux, and OS X), but this limitation will be removed in the future.

### 4.2. Comparison to Existing Approaches

Accelerating neural network simulations with the parallelization capabilities of GPUs has been a promising approach for more than a decade. The Brian2CUDA simulator presented here, builds on the foundations laid by these earlier simulators. For example, Brian2CUDA's spike propagation algorithm groups synapses based on their pre- and postsynaptic targets, as well as their delays into synapse *bundles*, similar to the approach of the NEMO simulator (Fidjeland et al., 2009; Fidjeland and Shanahan, 2010); in the case of homogeneous delays, Brian2CUDA's postsynaptic update algorithm results in a similar parallelization over synapses as in the dynamic parallelism approach described in Kasap and van Opstal (2018).

In recent years, several new, general-purpose simulators have seen the light of day, with each of them making different tradeoffs between the requirements of ease-of-use, flexibility and performance. To give a few recent examples: the Spike simulator (Ahmad et al., 2018) has been optimized for speed, but is implemented as a C++ library and therefore not easily useable for many researchers; the EDEN simulator (Panagiotou et al., 2021) runs arbitrary NeuroML v2 models (Cannon et al., 2014), which means it inherits NeuroML's focus on multi-compartmental models but also its limitations with regard to networks of spiking neurons; the NeuronGPU simulator (Golosio et al., 2021) comes with a convenient Python interface, but implementing new models requires editing the C++ source code of the simulator.

The Brian2CUDA interface and its general approach is comparable to the ANNarchy simulator (Vitay et al., 2015) and the GeNN simulator (Yavuz et al., 2016) when used together with its PyGeNN interface (Knight and Nowotny, 2021). By being a fully-featured backend for the Brian simulator, however, Brian2CUDA provides additional benefits for researchers that other simulators lack, such as a system of physical units, support for multi-compartmental models, and the possibility to precisely customize execution schedules. As we have shown in this article, Brian2CUDA not only provides flexibility and convenience, but also shows competitive performance for a wide range of network models.

The most similar approach to the Brian2CUDA package presented here is obviously the Brian2GeNN package, which is also implemented as a backend for the Brian simulator. Instead of generating CUDA code directly, the Brian2GeNN backend generates code for the GeNN simulator, which then in turn generates CUDA code. This approach has its advantages—e.g., Brian2GeNN will automatically benefit from performance optimizations in the GeNN package—but it also leads to a much more restricted set of Brian features that are supported. While the GeNN simulator provides a large amount of flexibility, it does not go as a far as Brian and Brian2CUDA, for example it does not allow for a customized execution order for all the elements of a simulation. The Brian2GeNN interface adds a number of additional restrictions. As a result, less common synapse implementations, in particular those that need access to and change variables both on the pre- and post-synaptic side, might not be supported. Brian2GeNN is also behind in enabling features added in newer versions of GeNN. Most importantly, GeNN added support for heterogeneous synaptic delays with its version 3.2, but this support is not yet available *via* the Brian2GeNN interface. The benchmark results for Brian2GeNN presented in this study should therefore be interpreted with caution and not necessarily be taken as indicative of GeNN's performance. For example, it appears as if the Brian2GeNN performance does not improve as much as expected when switching from double to single precision floats (Supplementary Figure S1), but that might be due to a suboptimal conversion of the model by Brian2GeNN.

## 5. Conclusion

By combining the flexibility of the Brian simulator with the simulation speed of GPUs, Brian2CUDA enables researchers to efficiently simulate spiking neural networks with minimal effort and thereby makes the advancements of GPU computing available to a larger audience of neuroscientists.

## Data availability statement

Publicly available datasets were analyzed in this study. The Brian2CUDA software package is publicly available on GitHub: https://github.com/brian-team/brian2cuda. The software version to reproduce the simulations in this study can be found at https://github.com/brian-team/brian2cuda/tree/paper2022 and instructions on how to run them can be found at https://github.com/brian-team/brian2cuda/tree/paper2022/brian2cuda/tools/benchmarking.

## Author contributions

MA conceptualized and supervised the project. DA and MA designed the Brian2CUDA algorithms and designed the benchmarks. DA developed the Brian2CUDA software and performed and analyzed the benchmarks, and wrote the initial draft of the manuscript. MS supervised the integration with Brian and contributed necessary features to Brian itself. DA, MS, and MA revised the manuscript. All authors contributed to the article and approved the submitted version.

## Funding

This work was supported by the Deutsche Forschungsgemeinschaft (DFG) in the framework of collaborative research centers SFB910 and SFB1315 (project number 327654276), the Open Access Publication Fund of TU Berlin and by Programme Investissements d'Avenir IHU FOReSIGHT (ANR-18-IAHU-01).

## Conflict of Interest

The authors declare that the research was conducted in the absence of any commercial or financial relationships that could be construed as a potential conflict of interest.

## Publisher's Note

All claims expressed in this article are solely those of the authors and do not necessarily represent those of their affiliated organizations, or those of the publisher, the editors and the reviewers. Any product that may be evaluated in this article, or claim that may be made by its manufacturer, is not guaranteed or endorsed by the publisher.

## Footnotes

^1^Note that while its Python package is named “brian2,” we will use the name “Brian” in this paper for simplicity.

^2^Note that using separate kernels allows us to support Brian's flexible execution scheduling, e.g., synaptic effect application between threshold detection and reset.

^3^In a population of neurons firing at 1 s-1 and a simulation time step of Δ*t* = 0.1 ms, on average only 0.01 % of the neurons spike at each time step.

^4^https://github.com/brian-team/brian2cuda/tree/paper2022/brian2cuda/tools/benchmarking.

^5^ https://github.com/brian-team/brian2cuda/tree/paper2022
