# Supplementary material for: Brian2CUDA: Flexible and Efficient Simulation of Spiking Neural Network Models on GPUs
Source: Front Neuroinform. 2022 Oct 31;16:883700. doi: 10.3389/fninf.2022.883700 (PMC9660315; doi:10.3389/fninf.2022.883700)
Supplement: Supplementary file 1 [file Data_Sheet_1.pdf]

Supplementary Material:

Brian2CUDA: Flexible and Efficient Simulation of Spiking Neural Network Models on GPUs

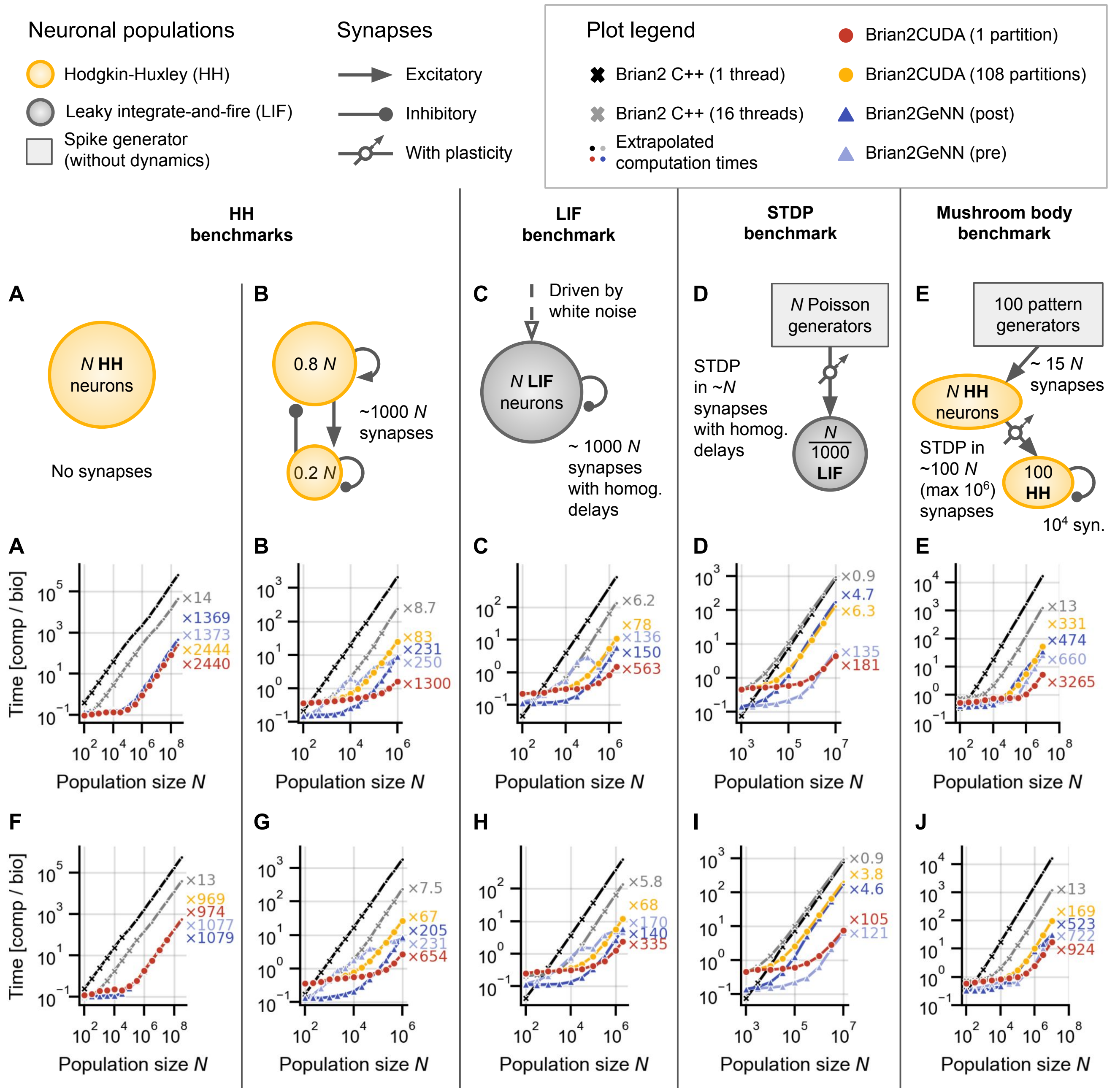

Figure S1. Additional benchmark results for networks without or with homogeneous delays. Same benchmarks as shown in figure 4, simulated with additional preferences for Brian2CUDA and Brian2GeNN. **(A) – (E)** Benchmarks simulated with Brian's single-precision preference for floating point numbers. Figure 5 shows the same data for a subset of the preferences shown here. **(F) – (J)** The same benchmarks simulated with Brian's double-precision preference for floating point numbers.

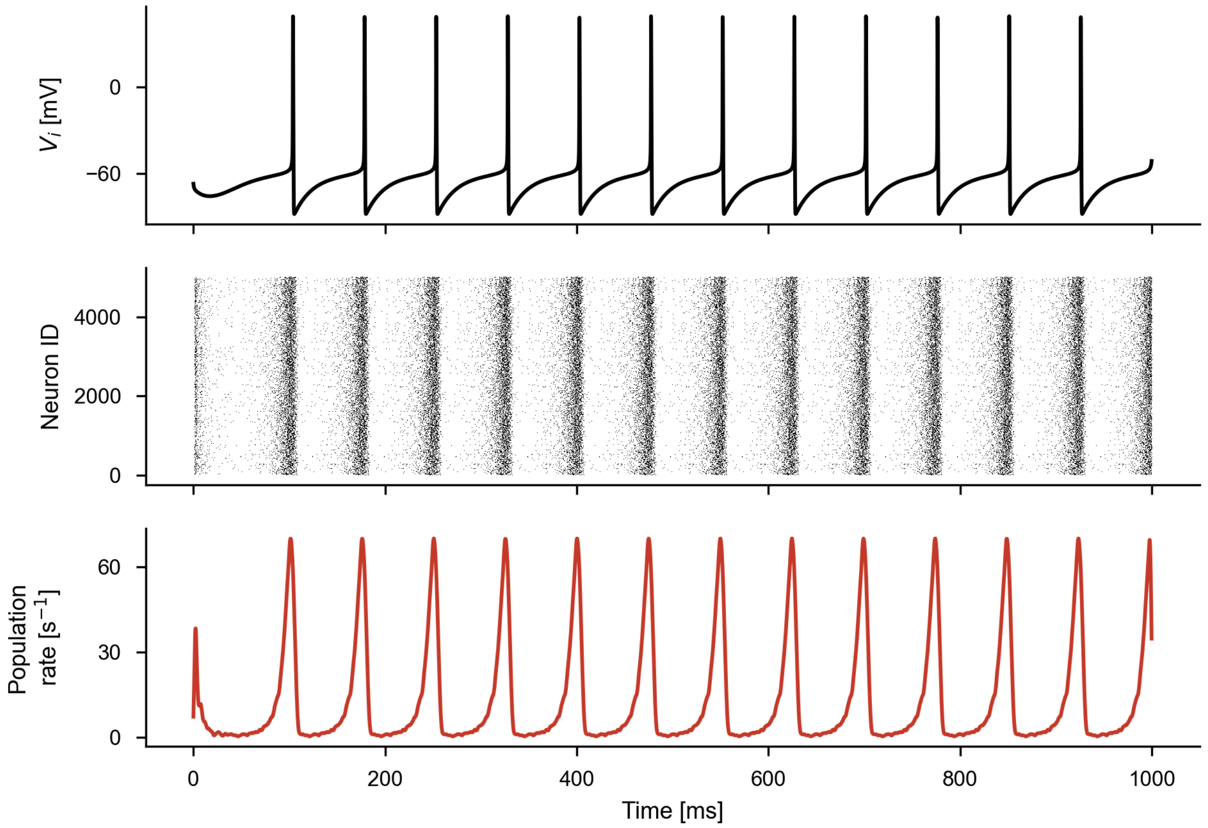

Figure S2. Example activity for the HH benchmark (see section 2.5.1.1) for an uncoupled population of 5000 neurons without synapses. Membrane potential trace for an example neuron (top); raster plot of the spiking activity for the full population (middle); population firing rate, smoothed with a Gaussian window with a standard deviation of 1 ms (bottom).

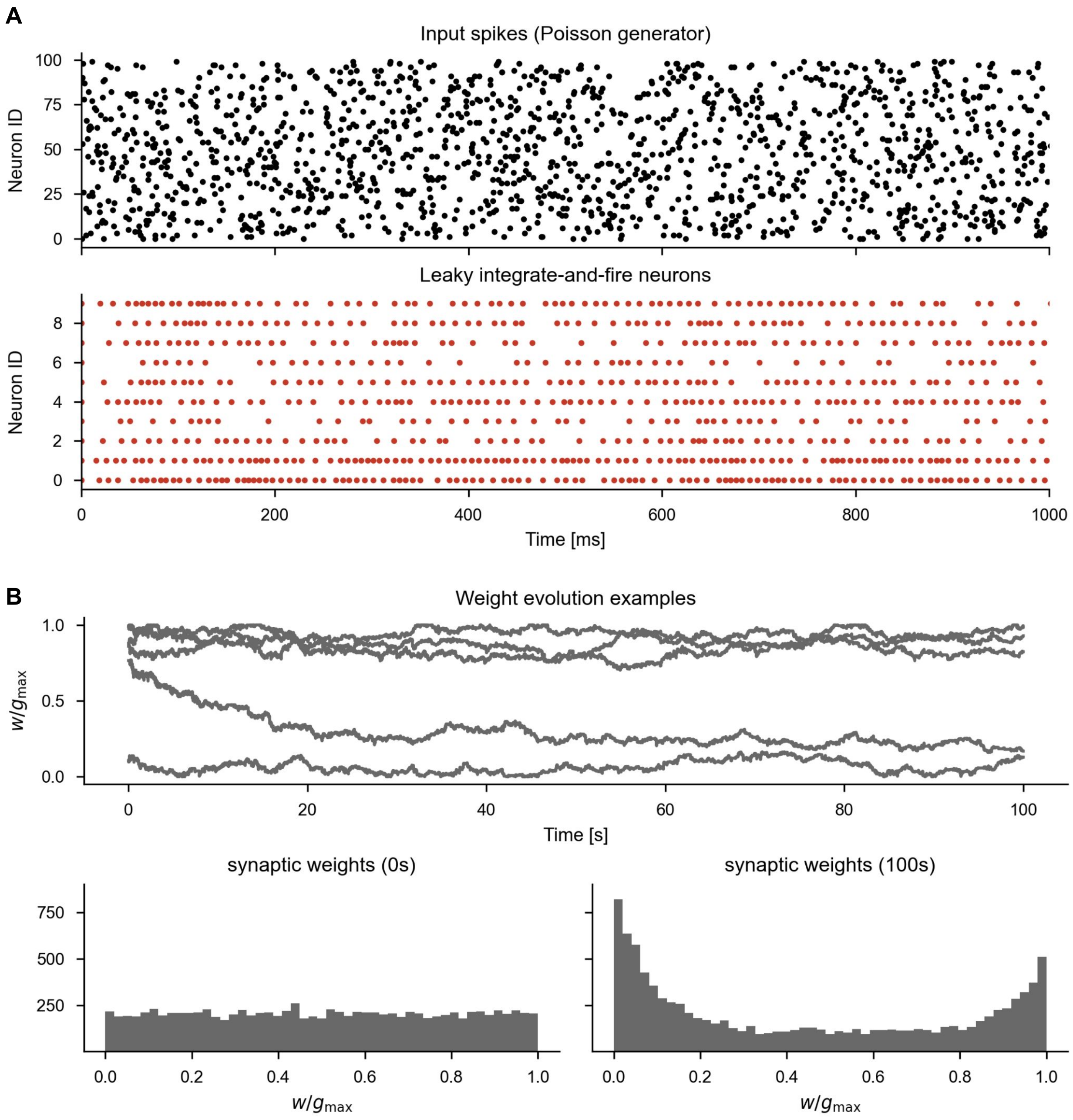

Figure S3. Example activity for the STDP benchmark (see section 2.5.1.3) with 10000 Poisson generators projecting to a population of 10 leaky integrate-and-fire neurons, with a connection probability of 10 % (i.e. each neuron receives on average input from 1000 Poisson generators). **(A)** Raster plot of the spiking activity of 100 Poisson generators (top) and all 10 integrate-and-fire neurons (bottom) during the first second of the simulation. **(B)** Synaptic weight evolution for five example weights over 100 s (top), and a comparison of the (normalized) weight distribution at the beginning of the simulation (bottom left) and after 100 s (bottom right).

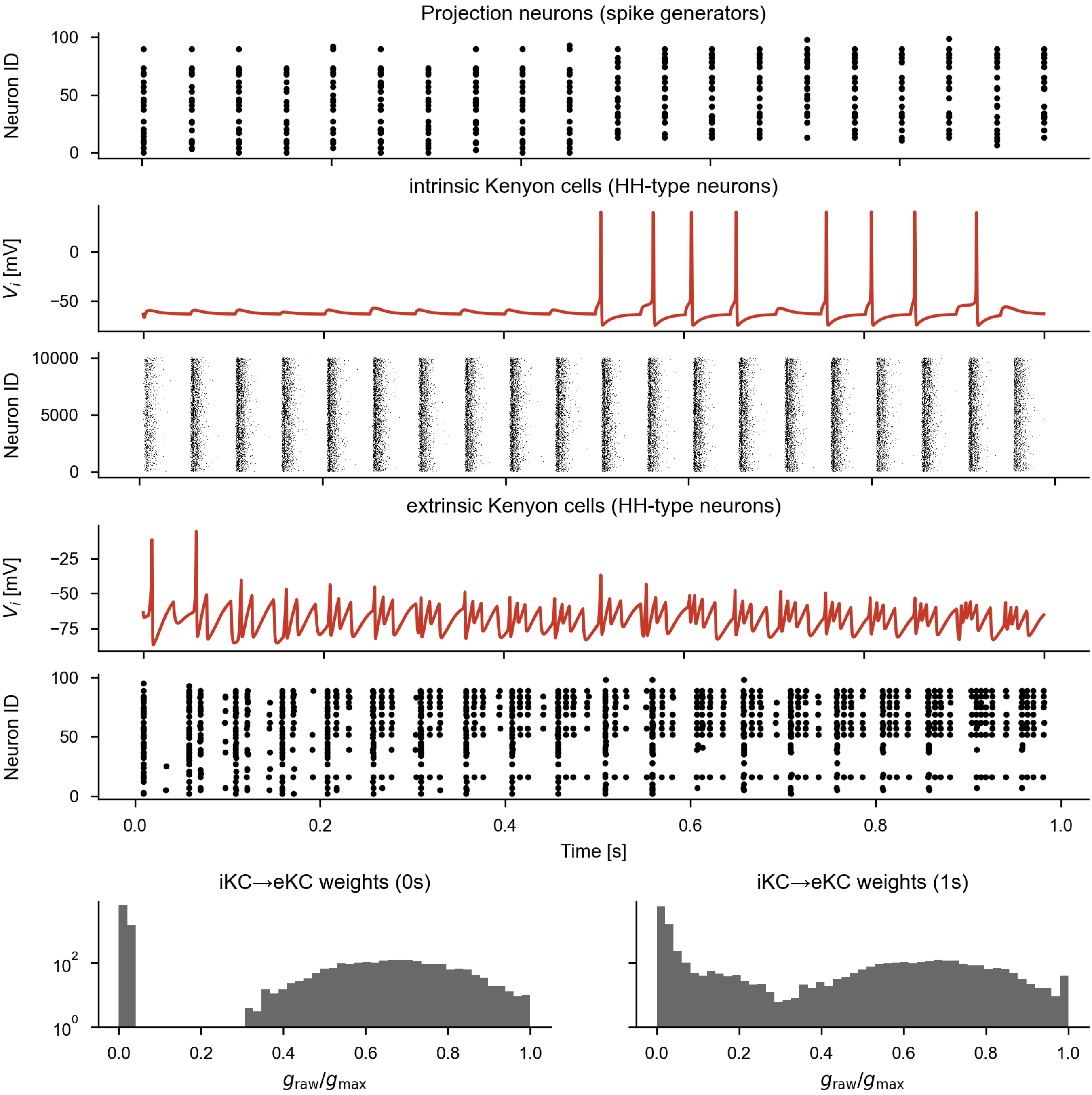

Figure S4. Example activity for the mushroom body benchmark (see section 2.5.1.4) with 100 projection neurons, 10000 intrinsic Kenyon cells, and 100 extrinsic Kenyon cells. Spiking activity of the projection neurons (top); membrane potential trace of an example intrinsic Kenyon cell (2nd row); spiking activity of the population of intrinsic Kenyon cells (3rd row); membrane potential trace of an example extrinsic Kenyon cell (4th row); spiking activity of the population of extrinsic Kenyon cells (5th row); comparison of the (normalized) weight distribution between intrinsic and extrinsic Kenyon cells at the beginning of the simulation (bottom left) and after 1 s (bottom right).
